# Discrete stochastic maximal regularity

**DOI:** 10.1007/s00208-026-03348-1

**Published:** 2026-02-13

**Authors:** Foivos Evangelopoulos-Ntemiris, Mark Veraar

**Affiliations:** Delft Institute of Applied Mathematics, Delft University of Technology, P.O. Box 5031, 2600 GA Delft, The Netherlands

**Keywords:** Primary 46N40, 60H15, Secondary 35B65, 42B37, 47D06, 60H35, 65J10, 65M12

## Abstract

In this paper, we investigate discrete regularity estimates for a broad class of temporal numerical schemes for parabolic stochastic evolution equations. We provide a characterization of discrete stochastic maximal $\ell ^p$-regularity in terms of its continuous counterpart, thereby establishing a unified framework that yields numerous new discrete regularity results. Moreover, as a consequence of the continuous-time theory, we establish several important properties of discrete stochastic maximal regularity such as extrapolation in the exponent *p* and with respect to a power weight. Furthermore, employing the $H^\infty$-functional calculus, we derive a powerful discrete maximal estimate in the trace space norm $D_A(1-\frac{1}{p},p)$ for $p \in [2,\infty )$.

## Introduction

Maximal $L^p$-regularity techniques play a central role in the theory of both deterministic and stochastic evolution equations of parabolic type (see the monographs [27, 50] and the surveys [4, 57], as well as the references therein). Although maximal regularity is inherently a linear concept, it becomes a powerful tool for analyzing nonlinear problems through linearization techniques. In particular, it allows us to establish local well-posedness and regularity results, and formulate sharp blow-up criteria for global existence for a wide class of equations.

In the deterministic setting, a theory of discrete maximal $\ell ^p$-regularity can already be found in [9] and was connected to discrete Fourier multiplier theory in [11, 12]. The connections between discrete maximal regularity and numerical analysis–specifically, the stability and convergence of numerical schemes for (non)linear PDEs–have been explored in numerous works (see, for example, [5–8, 31, 32, 34] and references therein), and this line of research remains highly active.

Motivated by these developments, we introduce a discrete analogue of stochastic maximal regularity. Our framework encompasses the exponential Euler method and a broad class of rational time discretization schemes, including the implicit Euler method. In addition to establishing new maximal regularity estimates, we derive discrete analogues of maximal estimates with optimal trace regularity. These results are expected to pave the way for improved convergence rates, as already observed in the deterministic setting. For instance, in the recent work [38], the authors used stochastic maximal regularity and non-optimal maximal estimates to prove pathwise uniform convergence estimates of a full discretization for the three-dimensional Allen-Cahn equation. The reader is also referred to [37, Remark 3.3] for an elementary example demonstrating the use of discrete stochastic maximal regularity in proving stability estimates.

The seminal results of [15] and [56] provide necessary and sufficient conditions for maximal $L^p$-regularity in the deterministic continuous-time context. In the stochastic setting, sufficient conditions involving the $H^\infty$-calculus of an operator *A* were established in [46, 48]. For a more detailed historical account on (stochastic) maximal regularity, we refer the reader to the recent survey [4] and the monograph [13].

Discrete versions of stochastic maximal regularity estimates are mainly known in a Hilbert space setting (see [19–21, 30]). An exception is [37], where a discrete stochastic maximal regularity is proved for the implicit Euler scheme in $L^q$-spaces using the boundedness of the $H^\infty$-calculus.

In our work, we establish a full theory of discrete stochastic maximal regularity, which in particular entails the following:

- An equivalence of the continuous and discrete setting for a large class of numerical schemes;
- Discrete stochastic maximal regularity results in Hilbert spaces, spaces like $L^q$, and real interpolation spaces;
- A discrete maximal estimate in the trace space $D_A(1-\frac{1}{p},p)$;
- All results are presented in a time-weighted setting.

Part (b) is a direct consequence of our equivalence theorem in (a) and the results of [4, 41, 46, 48] by the second author and his collaborators. In particular, (b) contains a far-reaching extension of the main result of [37]. The discrete maximal estimate mentioned in (c) is one of the deepest results of the paper, and has never been considered in a stochastic setting before. It incorporates maximal estimates and parabolic smoothing. As is well-known in the theory of stochastic processes, it can be rather delicate to prove maximal estimates (see [53]). Our proof relies on $H^\infty$-calculus, $\mathcal {R}$-boundedness techniques, fractional calculus, and deep results from interpolation theory.

### Formulation of our main results

Below, we will formulate some of the main results of the paper. For simplicity, we only present the unweighted setting in this subsection.

Let $X_0$ be (isomorphic to a closed subspace of) $L^q(\mathcal {O})$, for some $q\in [2, \infty )$, over a $\sigma$-finite measure space $(\mathcal {O}, \Sigma , \mu )$. Suppose that $X_1$ is a Banach space such that $X_1\hookrightarrow X_0$ densely and define the real and complex interpolation spaces

$$\begin{aligned} X_{\alpha ,p} :=(X_0, X_1)_{\alpha ,p} \ \ \text {and} \ \ X_{\alpha } :=[X_{0}, X_1]_{\alpha } , \ \ \ \alpha \in (0,1), \ p\in (1,\infty ). \end{aligned}$$

Throughout this manuscript, we impose the following assumption:

#### Assumption 1.1

1. *A* is a sectorial operator on $X_0$ of angle $\omega (A)<\frac{\pi }{2}$, and $D(A) = X_1$;
2. $R:[0,\infty )\rightarrow \mathcal {L}(X_0)$ is given by $R_{\tau } :=r(\tau A)$, where *r* is either the exponential function $r(z) :=e^{-z}$, or $r:\Sigma _{\theta }\rightarrow \mathbb {C}$ is an $A(\theta )$-stable rational function (i.e. $|r(z)|\le 1$ for $z\in \Sigma _{\theta }$) with $\theta \in (\omega (A),\tfrac{\pi }{2}]$ that is consistent of order $\ell \ge 1$ (i.e. $|r(z) - e^{-z}|\le Cz^{\ell +1}$ for $z\rightarrow 0$) and satisfies $r(\infty ) = 0$.

Here, $\Sigma _{\theta }:=\{z\in \mathbb {C}\setminus \{0\}:|\arg z|<\theta \}$. Given a time step $\tau >0$, let $t_n = n\tau$, $n \ge 0$, and consider the following discretization scheme: starting from $Y_0 :=0$, define recursively

1.1 $$\begin{aligned} Y_{n+1} :=R_\tau Y_{n} + R_\tau \int _{t_n}^{t_{n+1}} g(s) d W(s), \quad n\ge 0, \end{aligned}$$

where *W* is a cylindrical Brownian motion in $\ell ^2$ and $g\in L^p(\Omega ;L^p (0,T;\gamma (\ell ^2,X_{1/2})))$ is strongly progressively measurable.

The scheme *R* is said to have *discrete stochastic maximal* $\ell ^p$-*regularity* if there exists a constant *C*, independent of $\tau$ and *g*, such that

1.2 $$\begin{aligned} \mathbb {E}\sum _{n\ge 0} \tau \Vert A Y_n \Vert ^p_{X_0} \le C^p \mathbb {E}\Vert g\Vert _{L^p (0,T;\gamma (\ell ^2,X_{1/2}))}^p. \end{aligned}$$

We emphasize that the formulation (1.1) is sufficiently general to encompass various approximation methods. In particular, one can take $g(t) = g_n$ for $t\in (t_n, t_{n+1}]$, where $g_n\in L^p(\Omega ;\gamma (\ell ^2,X_{1/2}))$ is ${\mathscr {F}}_{t_n}$-measurable. This leads to the more common scheme

1.3 $$\begin{aligned} Y_{n+1} :=R_\tau Y_{n} + R_\tau g_n (W(t_{n+1}) - W(t_n)), \quad n\ge 0. \end{aligned}$$

In this case (1.2) becomes

$$ \mathbb {E}\sum _{n\ge 0} \tau \Vert A Y_n \Vert ^p_{X_0} \le C^p \mathbb {E}\sum _{n\ge 0} \tau \Vert g_n\Vert ^p_{\gamma (\ell ^2,X_{1/2}))}. $$

In most cases, $X_{1/2}$ is a (subspace of a) (fractional) Sobolev space $H^{s,q}(D)$, and in this situation

$$\begin{aligned} \gamma (\ell ^2,X_{1/2}) = H^{s,q}(D;\ell ^2). \end{aligned}$$

Our first main result is that this property can be characterized in the following way.

#### Theorem 1.2

(Characterization of discrete stochastic maximal $\ell ^p$-regularity) Let $q\in [2, \infty )$ and suppose that $X_0$ is isomorphic to a closed subspace of $L^q(\mathcal {O})$ with $(\mathcal {O}, \Sigma ,\mu )$ a $\sigma$-finite measure space. Suppose that Assumption 1.1 holds. Then for any $p\in [2, \infty )$ the following are equivalent:

1. *A* has stochastic maximal $L^p$-regularity;
2. *R* has discrete stochastic maximal $\ell ^p$-regularity.

As already mentioned, the continuous analogue in (1) was introduced in [2, 46, 48]. We will actually use the definition of [3, 4] where the space regularity is shifted by 1/2 as this is more natural in applications to SPDEs (see Remark 3.3 for further information).

Theorem 1.2 remains valid for any space $X_0$ satisfying certain geometric properties, specifically it holds if $X_0$ is UMD and has type 2. The strength of this result lies in the fact that it establishes discrete stochastic maximal regularity for a broad class of numerical schemes simultaneously. Moreover, it shows that it suffices to analyze the continuous-time setting, for which a well-developed theoretical framework already exists. Some of the immediate consequences for the discrete counterpart will be presented in Sect. 4.

The proof proceeds in several steps, detailed in Sects. 3.3, 3.4, and 3.5. Our approach not only establishes the equivalence in the stochastic setting but also suggests how similar equivalences could be obtained for other schemes in the deterministic setting. Indeed, in the deterministic case, such an equivalence was previously shown for the exponential Euler scheme in [29] and for the implicit Euler scheme in [31]. Our techniques can also be used to prove the equivalence for other schemes in the deterministic case.

An important consequence of Theorem 1.2 and a recent result in [4] is the following new result in the Hilbert space setting:

#### Corollary 1.3

(Discrete stochastic maximal $\ell ^p$-regularity for free). Suppose that $X_0$ is a Hilbert space and that Assumption 1.1 holds. Then *R* has discrete stochastic maximal $\ell ^p$-regularity for any $p\in [2, \infty )$.

In previous attempts, more structure was assumed on the operator *A* (e.g. variational structure or $H^\infty$-calculus). We show that this is not needed. The corresponding deterministic analogue also holds and has been established for various numerical schemes (see, for example, [12, Proposition 2.7]).

In addition to $\ell ^p$-estimates, it is often important to obtain parabolic $\ell ^\infty$-bounds for the sequence $(Y_n)_{n\ge 1}$. In the continuous-time setting, such *maximal estimates* are available (see [46]), where they are derived from optimal mixed space-time regularity results, which in turn rely on the $H^\infty$-calculus of the operator *A*. In the discrete case, we establish the following natural analogue of these maximal estimates.

#### Theorem 1.4

(Maximal estimate with parabolic regularization). Let $q\in [2, \infty )$ and suppose that $X_0$ is isomorphic to a closed subspace of $L^q(\mathcal {O})$ with $(\mathcal {O}, \Sigma ,\mu )$ a $\sigma$-finite measure space. Suppose that Assumption 1.1 holds, that *A* has a bounded $H^\infty$-calculus on $X_0$ of angle $<\pi /2$, and that $0\in \rho (A)$. Then for any $p\in (2, \infty )$, there is a constant *C* such that for every $g\in L^{p}_{\mathbb {F}}(\Omega ;L^p(\mathbb {R}_+;\gamma (\ell ^2,X_{1/2})))$ and every stepsize $\tau >0$,

$$\begin{aligned} \mathbb {E}\sup _{n\ge 1} \Vert Y_n \Vert _{X_{1-\frac{1}{p},p}}^p \le C^p \mathbb {E}\Vert g\Vert ^{p}_{L^p(\mathbb {R}_+;\gamma (\ell ^2,X_{1/2}))}, \end{aligned}$$

where $Y=(Y_n)_{n\ge 0}$ is given by (1.1).

It is important to note that $X_{1-\frac{1}{p},p}$ serves as the natural trace space associated with parabolic SPDEs. It is, in fact, the optimal space in which one can expect to obtain maximal estimates.

In the continuous-time setting, such estimates were proved using an optimal variant of the Da Prato Kwapień Zabczyk factorization method, combined with the operator-valued $$H^\infty $$-calculus, $$\mathcal {R}$$-boundedness of stochastic convolutions, and the analytical properties of the operator sum $$ d/dt + A$$. However, this approach does not carry over to the discrete setting, and a new argument is required. The key difference is that no natural identities or estimates for fractional derivatives in time are available in the discrete setting. To solve this, we embed the problem into a continuous-time setting by interpolating linearly and we estimate the fractional time derivatives by hand. To do this, many terms arise, all of which need a separate argument. Some of the leading-order terms are estimated through the operator-valued $$H^\infty $$-calculus and new $$\mathcal {R}$$-boundedness results for stochastic convolutions.

A natural question is whether Theorem 1.4 also holds in the case $p=2$. A key difference is that in this case there is no regularization. Indeed, if $X_0$ is a Hilbert space then $X_{1/2,2} = X_{1/2}$ (see [25, Corollary C.4.2]), so that the regularity space for the solution $(Y_n)_{n\ge 0}$ and the data *g* coincide. Still, the maximal estimate turns out to be true at least when $X_0$ is a Hilbert space. Remarkably, it is enough to assume that either $0\in \rho (A)$ *or* that the $H^\infty$-calculus is bounded.

#### Proposition 1.5

Let $X_0$ be a Hilbert space. Suppose that Assumption 1.1 holds with $\theta =\pi /2$. Suppose that $0\in \rho (A)$ or that *A* has a bounded $H^\infty$-calculus. Then there is a constant *C* such that for every $g\in L^{2}_{\mathbb {F}}(\Omega ;L^2(\mathbb {R}_+;\gamma (\ell ^2,X_{1/2})))$ and every stepsize $\tau >0$,

1.4 $$\begin{aligned} \mathbb {E}\sup _{n\ge 1} \Vert Y_n \Vert _{X_{1/2}}^2 \le C^2 \mathbb {E}\Vert g\Vert ^{2}_{L^2(\mathbb {R}_+;\gamma (\ell ^2,X_{1/2}))}. \end{aligned}$$

The assumption $r(\infty )=0$ is not used in the proof of Proposition 1.5.

### Overview

In Sect. 2 we present some essential mathematical background, covering sectorial operators, functional calculus, and key properties of numerical approximation schemes $R_\tau = r(\tau A)$, including crucial error estimates. We also review stochastic integration theory and the Burkholder–Davis–Gundy inequalities.

Section 3 formally defines discrete stochastic maximal $\ell ^p$-regularity, denoted by $\textrm{DSMR}(p,T)$. Our central achievement here is Theorem 3.6, establishing the equivalence between $\textrm{DSMR}$ for a scheme *R* and the continuous $\textrm{SMR}$ of the operator *A*, which directly yields new $\textrm{DSMR}$ results (e.g., Corollary 3.9). This section contains Theorem 1.2 as a special case.

In Sect. 4, we demonstrate that $\textrm{DSMR}$ inherits key permanence properties (such as *p*- and *T*-independence) from $\textrm{SMR}$ via Theorem 3.6. Notably, we also establish the equivalence of (discrete) stochastic maximal $\ell ^p$-regularity to a time-weighted formulation in Theorem 4.3.

Section 5 provides a critical technical tool for subsequent analysis. We establish the uniform $\mathcal {R}$-boundedness of relevant discrete stochastic convolution operator families, which is essential for proving the maximal estimates in Sect. 6.

The main result of Sect. 6 is Theorem 6.1 and contains the maximal estimate of Theorem 1.4 as a special case. This result is obtained by employing the $H^\infty$-functional calculus for the operator *A* and the $\mathcal {R}$-boundedness results from Sect. 5. A Hilbert space version for $p=2$ is also presented, and it particularly contains Proposition 1.5 as a special case.

### Notation

Let $w_{\alpha }(t) = t^{\alpha }$. For $p\in [1, \infty )$, a stepsize $\tau >0$, and a Banach space *Z*, let $\ell ^p_{\tau ,w_{\alpha }}(Z)$ denote the space of sequences $z:=(z_{j})_{j\ge 0}$ in *Z* such that

$$\begin{aligned} \Vert z\Vert _{\ell ^p_{\tau ,w_{\alpha }}(Z)} :=\Big (\sum _{n\ge 0} w_{\alpha }(t_{n+1}) \tau \Vert z_n\Vert ^p_Z\Big )^{1/p}<\infty , \end{aligned}$$

where $t_n = \tau n$ for $n\ge 1$. If a sequence $z = (z_n)_{n=0}^N$ consists of finitely many terms, we will use the same notation with the convention that $z_n = 0$ for $n\ge N+1$. For $T\in (0,\infty ]$ we write $L^p (0,T,w_{\alpha };Z)$ for the space of measurable functions $f:(0,T)\rightarrow Z$ such that

$$\begin{aligned} \Vert f\Vert _{L^p (0,T,w_{\alpha };Z)} :=\Big (\int _{0}^T \Vert f(t)\Vert _Z^p w_{\alpha }(t) dt\Big )^{1/p}<\infty . \end{aligned}$$

Whenever $\alpha =0$, we will omit the weight from the above notations.

For $a,b\in \mathbb {R}$ we will use the standard notation $a\lesssim b$ in case there is an (unimportant) constant *C* such that $a\le Cb$. Moreover, we write $a\eqsim b$ if $a\lesssim b$ and $b \lesssim a$. In case we want to emphasize that *C* depends on e.g. *p*, we write $a\lesssim _p b$ or $a \simeq _p b$.

For a Banach couple $(X_0, X_1)$, we use

$$\begin{aligned} X_{\alpha ,p} :=(X_0, X_1)_{\alpha ,p} \ \ \text {and} \ \ X_{\alpha } :=[X_{0}, X_1]_{\alpha } , \ \ \ \alpha \in (0,1), \ p\in (1,\infty ), \end{aligned}$$

for the real and complex interpolation spaces respectively.

The operator *A* will always be a sectorial operator on $X_0$ with domain $D(A) = X_1$. For $\theta \in (0,\pi )$, let $\Sigma _{\theta } :=\{z\in \mathbb {C}\setminus \{0\}:|\arg (z)|<\theta \}$ and let $\partial \Sigma _{\theta }$ denote its boundary oriented counterclockwise. The function $r:\Sigma _{\theta }\rightarrow \mathbb {C}$ denotes a rational function or $r(z)=e^{-z}$, and $R_{\tau } :=r(\tau A)$.

The notation *W* is used to denote a cylindrical Brownian motion, and $I_g(t) :=\int _0^t g d W$ for the stochastic integral of *g*. We also write $\Delta _n I_g :=I_g(t_{n+1}) - I_g(t_{n})$.

## Preliminaries

### Sectorial operators, functional calculus, and interpolation

For details on sectorial operators, semigroup theory, and functional calculus, the reader is referred to [16, 22, 26, 27, 35]. We briefly recall some of the key concepts used throughout the paper. For details on interpolation theory the reader is referred to [25, 55] and we will rely on standard results on real and complex interpolation. See the notation subsection at the end of the introduction.

Let (*A*, *D*(*A*)) be a closed operator on a Banach space *X*. The operator *A* is called *sectorial* if the domain and the range of *A* are dense in *X* and there exists $\nu \in (0,\pi )$ such that $\sigma (A)\subseteq \overline{\Sigma _{\nu }}$, where $\Sigma _{\nu }:=\{z\in \mathbb {C}\setminus \{0\}:|\arg z|<\nu \}$, and there exists $C>0$ such that

2.1 $$\begin{aligned} |\lambda |\Vert (\lambda -A)^{-1}\Vert _{\mathcal {L}(X)}\le C, \ \ \lambda \in \mathbb {C}\setminus \overline{\Sigma _{\nu }}. \end{aligned}$$

The *angle of sectoriality* $\omega (A)\in [0,\pi )$ is defined as the infimum over all $\nu$ for which a *C* exists such that (2.1) holds.

If $\omega (A)<\pi /2$, then $-A$ generates a strongly continuous semigroup $(e^{-tA})_{t\ge 0}$, which extends to a bounded analytic function on a sector.

#### Functional calculus

Let $H^1(\Sigma _{\theta })$ be the set of all holomorphic functions $f:\Sigma _{\theta }\rightarrow \mathbb {C}$ such that

$$\begin{aligned} \Vert f\Vert _{H^1(\Sigma _{\theta })} :=\sup _{|\phi |<\theta } \int _{0}^\infty |f(s e^{i\phi })| \frac{ds}{s}<\infty . \end{aligned}$$

Moreover, $H^\infty (\Sigma _{\theta })$ is the space of bounded holomorphic functions $f:\Sigma _{\theta }\rightarrow \mathbb {C}$ equipped with the supremum norm.

If *A* is a sectorial operator of angle $\omega (A)\in [0,\pi )$ and $f\in H^1(\Sigma _{\theta })$ with $\theta \in (\omega (A),\pi )$ then one can define the bounded operator *f*(*A*) by the Dunford integral (contour oriented counterclockwise)

$$\begin{aligned} f(A) :=\frac{1}{2\pi i} \int _{\partial \Sigma _{\nu }} f(z) (z-A)^{-1} \,dz, \end{aligned}$$

where $\nu \in (\omega (A),\theta )$ is chosen arbitrarily (see [26, Section 10.2]). Moreover, there is a constant $C=C(\theta ,A)$ such that

2.2 $$\begin{aligned} \sup _{t>0} \Vert f(tA)\Vert _{\mathcal {L}(X)} \le C \Vert f\Vert _{H^1(\Sigma _\theta )}. \end{aligned}$$

The above $H^1$-calculus is useful and can be used for any sectorial operator. In many important cases one can even prove that for all $\nu \in (\omega (A),\theta )$ and for every $f\in H^1(\Sigma _{\nu }) \cap H^\infty (\Sigma _{\nu })$ one has

2.3 $$\begin{aligned} \Vert f(A)\Vert _{\mathcal {L}(X)}\le C\Vert f\Vert _{H^\infty (\Sigma _{\nu })}. \end{aligned}$$

In this case we say that *A* has a *bounded* $H^\infty$-*calculus*. One can show that (2.3) uniquely extends to all $f\in H^\infty (\Sigma _{\nu })$. The infimum over all possible $\nu$ is called *the angle of the* $H^\infty$-*calculus*.

In the paper, we will use the $H^\infty$-calculus as a black box to prove several new results. By now, large classes of sectorial operators *A* are known to have a bounded $H^\infty$-calculus. One could even say that on $L^q$-spaces the counterexamples are typically only rather academic. A comprehensive list of examples can be found in the notes of [26, Chapter 10].

A general class of operators with a bounded $H^\infty$-calculus, in the case *X* is a Hilbert space, is given by the following: all operators *A* for which $-A$ generates a contraction semigroup on *X*. In this case, $\omega (A)$ coincides with the angle of the $H^\infty$-calculus. For details the reader is referred to [26, Theorems 10.2.24 and 10.4.21].

Another class can be given on $X = L^q$ for $q\in (1, \infty )$: all operators *A* for which $-A$ generates a positive contraction semigroup on *X*. In this case, one obtains that the angle of the $H^\infty$-calculus is $\le \pi /2$. Moreover, if *A* is also sectorial of angle $\omega (A)<\pi /2$, then one obtains that the angle of the $H^\infty$-calculus is $<\pi /2$ as well (although the two angles might differ). Details can be found in [26, Theorems 10.7.12 and 10.7.13].

#### Standard estimates for semigroups

Let (*A*, *D*(*A*)) be a sectorial operator of angle $<\pi /2$ on *X*. It is well-known that $-A$ generates a strongly continuous bounded analytic semigroup $(e^{-zA})_{z\in \Sigma _{\sigma }}$ for some $\sigma >0$ (see [26, Appendix G]). Moreover, letting $\lambda >0$, we can apply (2.2) to $f(z) = z^{\lambda } e^{-z}$, which is in $H^1(\Sigma _{\nu })$ for any $\nu <\pi /2$, to obtain

$$\begin{aligned} \sup _{t>0}\Vert (t A)^{\lambda } e^{-tA}\Vert _{\mathcal {L}(X)}<\infty . \end{aligned}$$

This implies that, for any $\varepsilon >0$, $\sup _{t>0}\Vert t^{\varepsilon } A^{1+\varepsilon } e^{-tA}\Vert _{\mathcal {L}(D(A),X)}<\infty$. Interpolating the two bounds gives that there is an $M\ge 0$ such that

2.4 $$\begin{aligned} \Vert A^{1+\varepsilon } e^{-tA}\Vert _{\mathcal {L}(V,X)} \le \frac{M}{t^{\frac{1}{2}+\varepsilon }}, \ t>0, \end{aligned}$$

where *V* is any Banach space that is continuously embedded into $(X, D(A))_{1/2,\infty }$. For example, one can take $V=D(A^{1/2})$, $V = [X, D(A)]_{1/2}$ or $V = (X, D(A))_{1/2,q}$ with $q\in [1, \infty ]$.

Another standard bound for a bounded strongly continuous semigroup is

2.5 $$\begin{aligned} \Vert e^{-tA} - e^{-sA}\Vert _{\mathcal {L}(V, X)} \le M (t - s)^{\alpha }, \end{aligned}$$

where $\alpha \in [0,1]$ and *V* is as above. Indeed, for $V= D(A)$ this holds for $\alpha =1$ and follows by writing $e^{-sA} - e^{-tA}=\int _s^t Ae^{-rA} dr$ on *D*(*A*), and using that $\sup _{r>0}\Vert e^{-rA}\Vert _{\mathcal {L}(X)}<\infty$. For $V = X$ the bound (2.5) holds with $\alpha =0$. The general case follows by interpolation.

#### Fractional powers

For a sectorial operator the fractional power $A^{z}$ can be defined for any $z\in \mathbb {C}$ via the so-called extended functional calculus (see [22, Chapter 3] and [27, Chapter 15]). In general, these operators are again closed unbounded operators.

The operator *A* is said to have *bounded imaginary powers (BIP)* if $A^{it}$ extends to a bounded operator on *X* for any $t\in \mathbb {R}$. In particular, this holds if *A* has a bounded $H^\infty$-calculus. Indeed, one can apply the calculus to the holomorphic and bounded function $z\mapsto z^{it}$. An important consequence of BIP is the following identification of the domains of the fractional powers and the complex interpolation spaces.

##### Lemma 2.1

Suppose that *A* is a sectorial operator and that *A* has BIP. Then for all $\theta \in (0,1)$,

$$\begin{aligned} D(A^{\theta }) = [X, D(A)]_{\theta } \end{aligned}$$

with equivalent norms.

A proof can be found in [22, Theorem 6.6.9], [27, Theorem 15.3.9], [55, 1.15.3].

### Approximation schemes

Let *X* be a Banach space, *A* a sectorial operator on *X* with angle $\omega (A)<\frac{\pi }{2}$ and let $(e^{-t A})_{t \ge 0}$ be the bounded analytic strongly continuous semigroup generated by $-A$. An approximation scheme is a strongly continuous function $R:[0,\infty )\rightarrow \mathcal {L}(X)$ with $R(0)=I$ that approximates the semigroup in the sense that there are $\alpha >0$ and a Banach space *V* with $V\hookrightarrow X$ densely such that

2.6 $$\begin{aligned} \Vert R_ \tau ^n - e^{-n\tau A} \Vert _{\mathcal {L}(V,X)} \le C \tau ^a, \qquad n\ge 0,\tau >0, \end{aligned}$$

where we set $R_\tau :=R(\tau )$ and $C>0$ is a constant independent of $n,\tau$. A common choice for *V* is the fractional domain $V=D(A^\alpha )$. An approximation is called stable if there is a constant $C>0$ such that

2.7 $$\begin{aligned} \Vert R_\tau ^n \Vert _{\mathcal {L}(X)} \le C, \quad n\ge 0, \tau >0. \end{aligned}$$

Typical examples of stable approximation schemes are the exponential Euler method $R_\tau = e^{-\tau A}$, the implicit Euler method $R_\tau :=(1+\tau A)^{-1}$, the Crank-Nicolson method $R_\tau :=(1-\frac{1}{2} \tau A)(1+\frac{1}{2} \tau A)^{-1}$ and, in general, any rational scheme $R_\tau :=r(\tau A)$ where $r:\Sigma _\theta \rightarrow \mathbb {C}$ is a rational function that is consistent and $A(\theta )$-stable with $\theta \in (\omega (A),\frac{\pi }{2}]$ (see [54, Theorems 9.1 and 9.2]). Recall that a rational function *r* is called *consistent of order* $\ell \ge 1$ if $|r(z)-e^{-z}| \le C|z|^{\ell +1}$ as $z \rightarrow 0$, and $A(\theta )$-stable if $|r(z)|\le 1$ for $z \in \Sigma _\theta$. Note that if *r* is $A(\theta )$-stable with $\theta \in (\omega (A), \frac{\pi }{2}]$ we can write

$$\begin{aligned} r(z) = \gamma + \sum _{k=1}^K \sum _{j=1}^J \gamma _{j,k} (a_j+z)^{-k}, \end{aligned}$$

where $\gamma = r(\infty )$, $K,J \in \mathbb {N}$ and the poles $-a_j \notin \Sigma _\theta$. Hence, we can define

2.8 $$\begin{aligned} r(\tau A) :=\gamma I + \sum _{k=1}^K \sum _{j=1}^J \gamma _{j,k} (a_j+ \tau A)^{-k}, \quad \tau >0, \end{aligned}$$

which is a bounded operator on *X* since $-a_j/\tau \in \rho (A)$.

We focus on the exponential Euler scheme $R_\tau = e^{-\tau A}$ and on rational schemes $R_\tau =r(\tau A)$ with the extra condition that $r(\infty )=0$ since they possess stronger convergence and stability properties (see Lemma 2.3). This excludes the Crank-Nicolson scheme, but includes the commonly used implicit Euler scheme, which is consistent of order $\ell =1$ and $A(\pi /2)$-stable, and the sub-diagonal Padé approximation schemes given by the rational functions $r_{n,n+1}=P_n/Q_{n+1}$ and $r_{n,n+2}=P_n/Q_{n+2}$ $(n\ge 1)$, where

$$\begin{aligned} P_n(z) :=\sum _{j=0}^{n} \frac{(n+m-j)! \, n!}{(n+m)! \, j! \, (n-j)!} (-z)^j, \qquad Q_m(z) :=\sum _{j=0}^{m} \frac{(n+m-j)! \, m!}{(n+m)! \, j! \, (m-j)!} z^j, \end{aligned}$$

which are consistent of order $\ell =2n+1$ and $\ell = 2n+2$ respectively, $A(\pi /2)$-stable, and satisfy $r_{n,n+1}(\infty )=r_{n,n+2}(\infty )=0$ (see [23, Theorem 4.12]). Another example is the Padé approximation scheme given by $r_{0,3}(z)=(1+z+z^2/2!+z^3/3!)^{-1}$, which is consistent of order $\ell =3$, $A(\theta )$-stable with $\theta \le 88.23^\circ$ and satisfies the extra condition $r(\infty )=0$ (see [23]).

The following lemma provides powerful estimates for such rational functions *r*, and can be found in [54, Lemmas 9.4 and 9.5].

#### Lemma 2.2

Let $\theta \in (0,\pi /2]$. Let $r:\Sigma _{\theta }\rightarrow \mathbb {C}$ be a rational function that is consistent of order $\ell \ge 1$ and $A(\theta )$-stable, and suppose that $r(\infty ) = 0$. Let $\nu \in (0,\theta )$. Then there exist constants $c=c(r,\nu )$ and $C=C(r,\theta )$ such that for all $z\in \Sigma _{\nu }$ and $n\ge 1$,

2.9 $$\begin{aligned} |r(z)^n - e^{-nz}|&\le C n |z|^{\ell +1} e^{-cn|z|},  &   \quad |z|\le 1, \end{aligned}$$

2.10 $$\begin{aligned} |r(z)|^n&\le C e^{-cn|z|},  &   \quad |z|\le 1, \end{aligned}$$

2.11 $$\begin{aligned} |r(z)|^n&\le C |z|^{-1} e^{-cn},  &   \quad |z|\ge 1. \end{aligned}$$

Moreover, one can take $c \le \cos \nu$ so that $|e^{-z}| \le e^{-c|z|}$ for $z\in \Sigma _{\nu }$.

A key estimate needed in our proofs is an improvement of (2.6) and (2.7):

#### Lemma 2.3

Let $R_{\tau } = r(\tau A)$, where *r* is either the exponential function $r(z) = e^{-z}$, or $r:\Sigma _{\theta }\rightarrow \mathbb {C}$ is a rational function which is consistent of order $\ell \ge 1$ and $A(\theta )$-stable with $\theta \in ( \omega (A), \frac{\pi }{2}]$, and $r(\infty ) = 0$. Then, there is a constant $C>0$ that depends on $r,\theta$ and *A* such that for every $\alpha \in [-1,1]$,

2.12 $$\begin{aligned} \Vert A^\alpha (e^{-\tau nA} -R_{\tau }^n)\Vert _{\mathcal {L}(X)}&\le C \frac{1}{ \tau ^{\alpha }n^{\ell +\alpha }},  &   \quad n \ge 1, \ \tau >0, \end{aligned}$$

2.13 $$\begin{aligned} \Vert A^\alpha R_{\tau }^n\Vert _{\mathcal {L}(X)}&\le C \frac{1}{\tau ^{\alpha }n^\alpha },  &   \quad n \ge 1, \ \tau >0. \end{aligned}$$

The estimate (2.12) was proved in [24, Theorem 3.1] for $\alpha =1$ and $\ell \ge 2$. The estimate (2.12) for $\alpha \in [-1,0]$ is already known, and can be found in [10, Theorem 5.1]. There it is even presented for general $\alpha \in [-\ell -1,0]$ and with the condition $r(\infty ) = 0$ replaced by $|r(\infty )|<1$. However, one can always reduce to $r(\infty ) = 0$ by a standard trick (see [36, Theorem 4.4]).

#### Proof

Note that (2.13) is immediate from (2.12) and the estimate $\sup _{t>0} t^{\alpha }\Vert A^{\alpha } e^{-t A}\Vert \le C$.

Let $f_{n}(z) :=z^\alpha \big ( e^{-n z} - r(z)^{n}\big )$. Note that by (2.2), in order to prove (2.12), it suffices to establish the estimate $\Vert f_{n}\Vert _{H^1(\Sigma _\theta )}\le C \frac{1}{ n^{\ell +\alpha }}$. To this end, let $\nu \in (\omega (A),\theta )$. By (2.9), there are constants $C=C(r,\theta )$ and $c=c(r,\nu )$ such that for every $|z| \le 1$ in the sector $\Sigma _{\nu }$,

$$\begin{aligned} |e^{-nz} -r( z)^{n}| \le C n | z|^{\ell +1} e^{-c n| z|} \end{aligned}$$

and consequently,

$$\begin{aligned} |f_{n}(z)| \le C n|z|^{\ell +1+\alpha } e^{-c n|z|} . \end{aligned}$$

Hence,

2.14 $$\begin{aligned} \int _{0}^{1} |f_{n} (\rho e^{\pm \nu i})| \frac{d\rho }{\rho }&\le C n \int _{0}^{1} \rho ^{\ell +\alpha } e^{-c n\rho } \, d\rho \nonumber \\&\le C n \int _{0}^{\infty } \rho ^{\ell +\alpha } e^{-c n\rho } \, d\rho \nonumber \\&= C c^{-(\ell +1+\alpha )} \Big (\frac{1}{ n}\Big )^{\ell +\alpha } \int _0^\infty s^{\ell +\alpha } e^{-s} \, ds = \widetilde{C} \frac{1}{ n^{\ell +\alpha }}. \end{aligned}$$

In the case where $\alpha <1$, by (2.11) we have that for $|z| \ge 1$ in the sector $\Sigma _{\nu }$,

$$\begin{aligned} |r( z)^{n} | \le C | z|^{-1} e^{-c n}. \end{aligned}$$

Therefore, by the triangle inequality, and noting that $|e^{-z}| \le e^{-c|z|}$ for $z\in \Sigma _{\nu }$,

$$\begin{aligned} |f_n(z)|\le C |z|^{\alpha } e^{-cn|z|} + C|z|^{-1+\alpha } e^{-cn}. \end{aligned}$$

Consequently,

$$\begin{aligned} \int _{1}^\infty |f_{n}(\rho e^{\pm \nu i})| \frac{d\rho }{\rho }&\le C \int _{1}^\infty \frac{1}{\rho ^{1-\alpha }} e^{-c n\rho } \, d\rho + C e^{-cn} \int _{1}^\infty \frac{1}{\rho ^{2-\alpha }}\,d\rho \\&\le C \int _{1}^\infty e^{-c n\rho } \, d\rho + C (1-\alpha )^{-1} e^{-cn} \le \widetilde{C} \frac{1}{n^{\ell +\alpha }}. \end{aligned}$$

This shows that

$$\begin{aligned} \Vert f_{n}\Vert _{H^1(\Sigma _\theta )} :=\sup _{\nu \le \theta } \int _0^\infty |f_{n}(\rho e^{\pm \nu i})| \frac{d\rho }{\rho } \le \widetilde{C} \frac{1}{ n^{\ell +\alpha }}. \end{aligned}$$

Suppose now that $\alpha =1$. Note that $r(\infty )=0$ and, by the maximum modulus principle, $|r(z)|<1$ on the interior of $\Sigma _\theta$. Hence, there are constants $c=c(r,\nu )$ and $C=C(r,\nu )$ such that $|r(z)|^2 \le C |z|^{-2}$ and $|r(z)|\le e^{-c}$ for $|z|\ge 1$ in the sector $\Sigma _\nu$. Therefore, for $n\ge 2$ and $z \in \Sigma _\nu$ with $|z|\ge 1$,

$$\begin{aligned} |r(z)|^n \le C|z|^{-2} e^{-c(n-2)}. \end{aligned}$$

This implies that

$$\begin{aligned} |f_{n}(z)| \le |z| e^{-c n|z|} + C|z|^{-1} e^{-c(n-2)} \end{aligned}$$

and consequently

$$\begin{aligned} \int _{1}^\infty |f_{n}(\rho e^{\pm \nu i})| \frac{d\rho }{\rho }&\le \int _{1}^\infty e^{-c n\rho } \, d\rho + C e^{-c(n-2)} \int _{1}^\infty \frac{1}{\rho ^{2}}\,d\rho \le \widetilde{C} \frac{1}{n^{\ell +1}}. \end{aligned}$$

This, together with (2.14), show that for $n \ge 2$,

$$\begin{aligned} \Vert f_{n}\Vert _{H^1(\Sigma _\theta )} :=\sup _{\nu \le \theta } \int _0^\infty |f_{n}(\rho e^{\pm \nu i})| \frac{d\rho }{\rho } \le C \frac{1}{ n^{\ell +1}}. \end{aligned}$$

For the case $n=1$, by the triangle inequality and since $\sup _{t>0}\Vert (t A) e^{-t A}\Vert < \infty$, it suffices to show that $\Vert (\tau A) r(\tau A)\Vert \le C$ uniformly in $\tau$. By (2.8) and noting that $r(\infty )=0$ we estimate

$$\begin{aligned} \Vert (\tau A) r(\tau A)\Vert&= \Big \Vert \sum _{j=1}^{J}\gamma _{j,1} (\tau A) (a_j+\tau A)^{-1} + \sum _{k =2}^{K} \sum _{j= 1}^{J} \gamma _{j,k} (\tau A) (a_j+\tau A)^{-k} \Big \Vert \\&\le \sum _{j =1}^{J} |\gamma _{j,1}| \, \Vert (\tfrac{\tau }{a_j} A)(1+\tfrac{\tau }{a_j} A)^{-1}\Vert \\&\quad + \sum _{k=2}^K \sum _{j=1}^J |\gamma _{j,k}|\, \Vert (\tau A)(a_j+\tau A)^{-k}\Vert \le C \end{aligned}$$

by noting that $\sup _{t>0} \Vert (t A)(1+t A)^{-1}\Vert \le C$ and by applying (2.2) for $f_{k,j}(z)= z/(a_j+z)^k$ which belongs to $H^1(\Sigma _\nu )$ for $k \ge 2, j \ge 1$. $\square$

### Stochastic integration

In principle, there is a full analogue of stochastic integration theory in infinite dimensions. However, geometric conditions on the underlying spaces are required. In order to give a satisfactory explanation of stochastic integration in a Banach space setting, we need $\gamma$-radonifying operators $\gamma (H,X)$ where *H* is a Hilbert space and *X* a Banach space. For details we refer to [26, Chapter 9]. Specializing to Hilbert spaces *X*, this class of operators reduces to the Hilbert-Schmidt operators $\mathcal {L}_2(H,X)$. Moreover, in the important case $X = L^q(\mathcal {O})$, one can identify $\gamma (H,X)$ with $L^q(\mathcal {O};H)$.

Let $(\Omega ,{\mathscr {F}}, \mathbb {P})$ denote a probability space with filtration $\mathbb {F}=({\mathscr {F}}_t)_{t\ge 0}$.

#### Definition 2.4

Let *H* be a Hilbert space. A bounded linear operator $W:L^2(\mathbb {R}_+;H)\rightarrow L^2(\Omega )$ is said to be a *cylindrical Brownian motion* in *H* if the following are satisfied:

- for all $f\in L^2(\mathbb {R}_+;H)$ the random variable *W*(*f*) is centered Gaussian;
- for all $t\in \mathbb {R}_+$ and $f\in L^2(\mathbb {R}_+;H)$ with support in [0, *t*], *W*(*f*) is ${\mathscr {F}}_t$-measurable;
- for all $t\in \mathbb {R}_+$ and $f\in L^2(\mathbb {R}_+;H)$ with support in $[t,\infty ]$, *W*(*f*) is independent of ${\mathscr {F}}_t$;
- for all $f_1,f_2\in L^2(\mathbb {R}_+;H)$ we have $\mathbb {E}(W(f_1)W(f_2))=(f_1,f_2)_{L^2(\mathbb {R}_+;H)}$.

Given *W*, the process $t\mapsto W(\textbf{1}_{(0,t]} h)$ is a Brownian motion for each $h\in H$.

The way to think about *W* is that it is given by $t\mapsto \sum _{n\ge 1} W^n(t) h_n$, where $(W^n)_{n\ge 1}$ are independent standard $\mathbb {F}$-Brownian motions, and $(h_n)_{n\ge 1}$ an orthonormal basis for *H*. However, since the above series does not define an *H*-valued random variable, the definition is given in a weaker sense.

However, the following convergence property does hold: if $S\in \gamma (H,X)$, then

2.15 $$\begin{aligned} S W(t) :=\sum _{k\ge 1} W(t) h_k S h_k, \end{aligned}$$

where the convergence takes place in $L^p(\Omega ;X)$ for all $p\in [1, \infty )$.

A process $g :\mathbb {R}_+\times \Omega \rightarrow \mathcal {L}(H,X)$ is called *H*-*strongly progressively measurable* if for all $t\in [0,T]$, $g|_{[0,t]}$ is strongly $\mathcal {B}([0,t])\otimes {\mathscr {F}}_t$-measurable (where $\mathcal {B}$ denotes the Borel $\sigma$-algebra).

For $0\le a<b\le T$ and a strongly ${\mathscr {F}}_a$-measurable $\xi :\Omega \rightarrow \gamma (H,X)$, the stochastic integral of $\textbf{1}_{(a,b]} \xi$ is defined by

2.16 $$\begin{aligned} \int _0^{t} \textbf{1}_{(a,b]} \xi d W:=\xi (W(b\wedge t)-W(a\wedge t)), \end{aligned}$$

where the series can be shown to be convergent as in (2.15) by the independence of ${\mathscr {F}}_a$ and $W(b\wedge t)-W(a\wedge t)$.

The space $L^p_{\mathbb {F}}((0,T)\times \Omega ;\gamma (H,X))$ denotes the subspace of $L^p((0,T)\times \Omega ;\gamma (H,X))$ consisting of all strongly progressively measurable processes. It can be shown that this coincides with the closure of the adapted step processes of finite rank (see [45, Proposition 2.10]). Moreover, let $L^p_{\mathbb {F}}(\Omega ;\ell ^p_\tau (Z))$ denote the subspace of adapted elements in $L^p(\Omega ;\ell ^p_\tau (Z))$.

The following proposition provides a simple sufficient condition for stochastic integrability. For the definition of type 2, see [26, Chapter 7]. Spaces of type 2 include $L^q$, $W^{s,q}$, and $H^{s,q}$ etc. for $q\in [2, \infty )$ and $s\in \mathbb {R}$. The definition of UMD can be found in [25, Chapter 4]. Spaces with UMD include $L^q$, $W^{s,q}$, and $H^{s,q}$ with $q\in (1, \infty )$ and $s\in \mathbb {R}$.

#### Proposition 2.5

(One-sided Burkholder–Davis–Gundy inequality) Let *X* be a UMD Banach space with type 2. Then for every $p\in [0,\infty )$, the mapping $g\mapsto \int _0^\cdot g\,\text {d}W$ extends to a continuous linear operator from $L^p_{\mathbb {F}}(\Omega ;L^2(\mathbb {R}_+;\gamma (H,X)))$ into $L^p(\Omega ;C_b([0,\infty );X))$. Moreover, for $p\in (0,\infty )$ there exists a constant $C_{p,X}$ such that for all $g\in L^p_{\mathbb {F}}(\Omega ;L^2(\mathbb {R}_+;\gamma (H,X)))$, the following estimate holds

$$\begin{aligned} \textstyle \mathbb {E}\sup _{t\ge 0}\Big \Vert \int _0^t g(s)\,\text {d}W(s)\Big \Vert _{X}^p \le C_{p,X}^p \mathbb {E}\Vert g\Vert _{L^2(\mathbb {R}_+;\gamma (H,X))}^p. \end{aligned}$$

The above result is sharp for Hilbert spaces *X*. However, if *X* is not a Hilbert space, a more precise bound is available, which, though explicitly used only in Sect. 5, forms the crucial basis for the proofs of Theorem 1.4 and Corollary 3.9. For simplicity, we only formulate the result for $X = L^q(\mathcal {O})$. For details the reader is referred to [45].

#### Proposition 2.6

(Two-sided Burkholder–Davis–Gundy inequality) Let $X = L^q(\mathcal {O})$ with $q\in (1, \infty )$. Then for every $p\in [0,\infty )$, the mapping $g\mapsto \int _0^\cdot g\,\text {d}W$ extends to a continuous linear operator from $L^p_{\mathbb {F}}(\Omega ;L^q(\mathcal {O};L^2(\mathbb {R}_+;H))$ into $L^p(\Omega ;C_b([0,\infty ); L^q(\mathcal {O}))$. Moreover, for $p\in (0,\infty )$ and for all $g\in L^p_{\mathbb {F}}(\Omega ;L^q(\mathcal {O};L^2(\mathbb {R}_+;H)))$, the following two-sided estimate holds

$$\begin{aligned} \mathbb {E}\sup _{t\ge 0}\Big \Vert \int _0^t g(s)\,\text {d}W(s)\Big \Vert _{L^q}^p \eqsim _{p,q} \mathbb {E}\Vert g\Vert _{L^q(\mathcal {O};L^2(\mathbb {R}_+;H))}^p. \end{aligned}$$

Observe that for $q\in [2, \infty )$, by identifying $\gamma (H,L^q(\mathcal {O}))=L^q(\mathcal {O};H)$, and by Minkowski’s inequality,

$$\begin{aligned} L^2(\mathbb {R}_+;\gamma (H,L^q(\mathcal {O}))) = L^2(\mathbb {R}_+;L^q(\mathcal {O};H)) \hookrightarrow L^q(\mathcal {O};L^2(\mathbb {R}_+;H)), \end{aligned}$$

which explains why Proposition 2.6 gives a sharper estimate, which is even two-sided.

## Discrete stochastic maximal regularity

In this section, we introduce discrete stochastic maximal regularity for arbitrary schemes. A definition of the continuous variant is more standard and can be found in Definition 3.5. The main result of this section shows that under mild conditions on the scheme, discrete stochastic maximal $\ell ^p$-regularity and stochastic maximal $L^p$-regularity are equivalent. Since stochastic maximal $L^p$-regularity is known to hold for a large class of operators *A*, we immediately obtain a wide range of examples for the discrete setting.

From now on, we fix a probability space $(\Omega ,{\mathscr {F}}, \mathbb {P})$ with filtration $\mathbb {F} = ({\mathscr {F}}_{t})_{t\ge 0}$, and a cylindrical Brownian motion *W* with respect to $\mathbb {F}$ on *H*. Moreover, we suppose that $X_0$ and $X_1$ are UMD spaces with type 2 such that $X_1\hookrightarrow X_0$ densely. Let $X_{\alpha } :=[X_{0}, X_1]_{\alpha }$ for $\alpha \in (0,1)$ denote the complex interpolation spaces.

In the rest of this section we suppose that Assumption 1.1 is satisfied. By the assumptions on *A*, one sees that $-A$ generates a bounded analytic strongly continuous semigroup, which will be denoted by $(e^{-tA})_{t\ge 0}$. Moreover, the fractional powers $A^{\alpha }$ for $\alpha >0$ are well-defined.

Let $T \in (0,+\infty ]$. We say that $\tau >0$ is *admissible* if $T/\tau \in \mathbb {N}$ or $T =\infty$. For an admissible $\tau$ let $\pi _\tau :=\{t_n = n\tau :0 \le n \le N \}$ be a uniform partition of [0, *T*], where $N = T/\tau$ if $T<\infty$, and $N=\infty$ if $T = \infty$. For $g \in L^p_{\mathbb {F}}(\Omega ;L^p (0,T;\gamma (H,X_{1/2})))$, we define $Y=(Y_n)_{n=0}^N$ recursively by

3.1 $$\begin{aligned} {\left\{ \begin{array}{ll} Y_{n+1} :=R_\tau Y_{n} + R_\tau \Delta _n I_g, \quad n=0,\dots , N-1 \\ Y_0:=0. \end{array}\right. } \end{aligned}$$

Here we set $I_g :=\int _0^\cdot g(s) d W(s)$ and

$$\begin{aligned} \Delta _n I_g :=I_g(t_{n+1}) - I_g(t_{n}) = \int _{t_{n}}^{t_{n+1}} g(s) d W(s). \end{aligned}$$

Alternatively, we can write $Y_n$ as a discrete stochastic convolution

3.2 $$\begin{aligned} Y_n = \sum _{j=0}^{n-1} R_\tau ^{n-j} \Delta _j I_g , \quad n=1,\dots , N. \end{aligned}$$

### Definition and basic properties

The following definition is central in this section and describes a certain regularity property of $Y=(Y_n)_{n=0}^N$ as defined in (3.1).

#### Definition 3.1

(Discrete stochastic maximal regularity). Suppose that Assumption 1.1 holds. Let $T\in (0,+\infty ]$ and $p\in [2,+\infty )$. The scheme *R* is said to have *discrete stochastic* $\ell ^p$-*maximal regularity* on (0, *T*) if there is a constant $C>0$ such that, for every admissible $\tau >0$, every uniform partition $\pi _\tau :=\{t_n=n \tau :0\le n \le N\}$ of [0, *T*], for every $g \in L^p_{\mathbb {F}} (\Omega ; L^p (0,T;\gamma (H,X_{1/2})))$ the approximation scheme $Y=(Y_n)_{n=0}^N$ given in (3.1) belongs to $X_1$ a.s. and satisfies

3.3 $$\begin{aligned} \Vert A Y \Vert _{L^p(\Omega ;\ell ^p_\tau (X_{0}))} \le C \Vert g\Vert _{L^p(\Omega ;L^p (0,T;\gamma (H,X_{1/2})))}. \end{aligned}$$

The least admissible constant *C* is denoted by $C_{\textrm{DSMR}(p,T)}^R$. In case the above holds, we will write $R\in \textrm{DSMR}(p,T)$.

The constant *C* is allowed to depend on *T*, but should be uniform in the stepsize $\tau$. Note that by (3.2) and since $Y_0=0$, (3.3) is equivalent to

$$\begin{aligned} \Big ( \mathbb {E}\sum _{n=1}^{N-1} \tau \Big \Vert A \sum _{j=0}^{n-1} R_\tau ^{n-j} \, \Delta _j I_g \Big \Vert ^p_{X_0} \Big )^{1/p} \le C \big (\mathbb {E}\Vert g\Vert _{L^p (0,T;\gamma (H,X_{1/2}))}^p\big )^{1/p}. \end{aligned}$$

Some further properties of operators with discrete stochastic $\ell ^p$-maximal regularity are collected in Sect. 4.

#### Remark 3.2

1. It is always true that $Y_n\in X_1$ a.s. since we assume $r(\infty ) = 0$ (see (2.8)). If $r(\infty )\ne 0$ one does not have $Y_n\in X_1$ in general unless *A* is bounded.
2. One can replace the homogeneous norm $\Vert A Y \Vert _{L^p(\Omega ;\ell ^p_\tau (X_{0}))}$ in (3.3) with the inhomogeneous one $\Vert Y \Vert _{L^p(\Omega ;\ell ^p_\tau (X_{1}))}$. This leads to the following inhomogeneous version of discrete stochastic $\ell ^p$-maximal regularity:
  3.4 $$\begin{aligned} \Vert Y \Vert _{L^p(\Omega ;\ell ^p_\tau (X_{1}))} \le C \Vert g\Vert _{L^p(\Omega ;L^p (0,T;\gamma (H,X_{1/2})))}. \end{aligned}$$
  Clearly (3.4) implies (3.3) and the two are equivalent if $0 \in \rho (A)$. Furthermore, when restricted to finite time intervals $T<\infty$ one need not assume that $0 \in \rho (A)$. Indeed, by Proposition 2.5 and the stability result (2.7) we immediately obtain that
  $$\begin{aligned} \Vert Y\Vert _{L^p(\Omega ;\ell ^p_\tau (X_{1/2}))} \lesssim _{p,X_0,R} T^{\frac{1}{p}} \Vert g\Vert _{L^2(0,T;\gamma (H,X_{1/2}))} \le T^{\frac{1}{2}} \Vert g\Vert _{L^p(0,T;\gamma (H,X_{1/2}))}. \end{aligned}$$
  The same estimate holds if $X_{1/2}$ is replaced by $X_{\alpha }$ (for both *Y* and *g*) for any $\alpha \in [0,1]$.

#### Remark 3.3

In case *A* has bounded imaginary powers (BIP) and $0\in \rho (A)$, there are several equivalent ways to formulate discrete stochastic $\ell ^p$-maximal regularity, and the same applies to the continuous case, which will be defined below in Definition 3.5. In particular, BIP holds if *A* has a bounded $H^\infty$-calculus.

Under the assumption that *A* has BIP, $A^{1/2}$ defines an isomorphism from $X_{1/2}$ to $X_{0}$, so that one could define discrete stochastic $\ell ^p$-maximal regularity as

$$\begin{aligned} \Vert A^{\frac{1}{2}}Y \Vert _{L^p(\Omega ;\ell ^p_\tau (X_{0}))} \le C \Vert g\Vert _{L^p(\Omega ;L^p (0,T;\gamma (H,X_{0})))}, \end{aligned}$$

which is quite common. The advantage of (3.3) is that it aligns well with its deterministic variant, in which one has regularization from $X_0$ into $X_1$.

#### Remark 3.4

In the special case where $g :=\sum _{n=0}^{N-1} g_n \textbf{1}_{[t_n,t_{n+1})}$ with $g_n \in L^p(\Omega ,{\mathscr {F}}_{t_n},\mathbb {P}; \gamma (H,X_{1/2}))$, one can write

$$\begin{aligned} \Delta _n I_g = g_{n} \, \Delta W_{n}, \end{aligned}$$

where $\Delta _n W :=W(t_{n+1}) - W(t_{n})$ and we used (2.15). This case is a popular choice for the discrete setting as well. Note that in this situation

$$\begin{aligned} \Vert g\Vert _{L^p (\Omega ; L^p (0,T;\gamma (H,X_{1/2})))} = \Vert (g_n)_{n\ge 0}\Vert _{L^p(\Omega ;\ell ^p_\tau ( \gamma (H,X_{1/2})))}. \end{aligned}$$

### Equivalence of continuous and discrete $\textrm{SMR}$

The following definition is a special case of the definition of stochastic maximal regularity in [4].

#### Definition 3.5

(Continuous stochastic maximal regularity). Suppose that Assumption 1.1(1) holds. Let $T\in (0,+\infty ]$ and $p\in [2,+\infty )$. The operator *A* is said to have *stochastic* $L^p$-*maximal regularity* on (0, *T*) if there is a constant $C>0$ such that, for every $g \in L^p_{\mathbb {F}} (\Omega ; L^p (0,T;\gamma (H,X_{1/2})))$, the mild solution

3.5 $$\begin{aligned} y(t) :=\int _0^t e^{-(t-s)A} g(s) d W(s), \quad t\in (0,T), \end{aligned}$$

belongs to $X_1$ a.s. and satisfies

3.6 $$\begin{aligned} \Vert A y \Vert _{L^p(\Omega ;L^p(0,T;X_0))} \le C \Vert g\Vert _{L^p(\Omega ;L^p (0,T;\gamma (H,X_{1/2})))}. \end{aligned}$$

The least admissible constant *C* is denoted by $C_{\textrm{SMR}(p,T)}^A$. In case the above holds, we will write $A\in \textrm{SMR}(p,T)$.

Some basic properties of stochastic $L^p$-maximal regularity are collected in Proposition 4.1.

The main result of this section is the following.

#### Theorem 3.6

Suppose that Assumption 1.1 holds. Let $T\in (0,+\infty ]$ and $p \in [2,\infty )$. Then the following are equivalent:

1. *A* has stochastic maximal $L^p$-regularity on (0, *T*);
2. *R* has discrete stochastic maximal $\ell ^p$-regularity on (0, *T*).

Moreover, $C_{\textrm{SMR}(p,T)}^A \le C_{\textrm{DSMR}(p,T)}^R\le K (C_{\textrm{SMR}(p,T)}^A + 1)$, where the constant *K* depends on *p*, $X_0$, the function *r*, and on the sectoriality constant and angle of *A*.

**Fig. 1:**
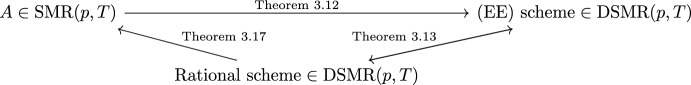
Flowchart of the proof of Theorem 3.6

The proof of the implication from $\textrm{SMR}$ to $\textrm{DSMR}$ will be given in Sects. 3.3 and 3.4 for the exponential Euler and rational schemes, respectively. The converse is simpler and will be proved in Sect. 3.5. See Fig. 1 for a flowchart of the proof of Theorem 3.6. Furthermore, in Theorem 4.3 we show that (1) and (2) are equivalent to their weighted counterparts.

#### Remark 3.7

(Quasi-uniform partitions). Theorem 3.6 extends to quasi-uniform partitions $\pi$ of [0, *T*], namely partitions that are given by variable time-steps $(\tau _n)_{n=1}^N$ for which there is a constant $\mu >0$ with $\mu ^{-1} \le \frac{\tau _{\max }}{\tau _{\min }} \le \mu ,$ where $\tau _{\max }$ and $\tau _{\min }$ denote the maximum and minimum time-step, respectively. Note that the constant *K* of Theorem 3.6 will also depend on $\mu$. In this case, the approximation scheme becomes

$$\begin{aligned} {\left\{ \begin{array}{ll} Y_{n+1} :=R_{\tau _{n+1}} Y_{n} + R_{\tau _{n+1}} \Delta _n I_g, \quad n=0,\dots , N-1 \\ Y_0:=0 \end{array}\right. } \end{aligned}$$

and the solution $(Y_n)$ is given by

3.7 $$\begin{aligned} Y_n = \sum _{j=0}^{n-1} R^{n,j}_\pi \Delta _j I_g , \quad n=1,\dots , N, \end{aligned}$$

where $(R^{n,j})_{j \le n}$ denotes the discrete evolution family given by

$$ R^{n,j}_\pi :={\left\{ \begin{array}{ll} R_{\tau _n} \cdots R_{\tau _{j+1}} & 0\le j \le n-1\\ I &  j=n. \end{array}\right. } $$

We say that *R* has discrete stochastic maximal $\ell ^p$-regularity if for every $g \in L^p_{\mathbb {F}} (\Omega ; L^p (0,T;\gamma (H,X_{1/2})))$ the approximation scheme $Y=(Y_n)_{n=0}^N$ given in (3.7) belongs to $X_1$ a.s. and satisfies

$$\begin{aligned} \Big ( \mathbb {E}\sum _{n=1}^{N-1} \tau _{n+1} \Big \Vert A \sum _{j=0}^{n-1} R^{n,j}_\pi \, \Delta _j I_g \Big \Vert ^p_{X_0} \Big )^{1/p} \le C \big (\mathbb {E}\Vert g\Vert _{L^p (0,T;\gamma (H,X_{1/2}))}^p\big )^{1/p}. \end{aligned}$$

The constant *C* does not depend on *g* and $\pi$ but it is allowed to depend on $\mu$. Since uniform partitions are quasi-uniform, Theorem 3.17 proves the implication (2) $\implies$ (1) of Theorem 3.6 for quasi-uniform partitions. In order to prove the converse implication, it suffices to check that Theorems 3.12 and 3.13 extend to quasi-uniform partitions (see Fig. 1). Note that the proof of Theorem 3.12 mainly relies on establishing (3.10) and the latter follows from estimating $\Vert \Phi (t,s,n,j)\Vert ^2_{\mathcal {L}(X_{1/2},X_0)} \lesssim [\tau (n-j-1)^2]^{-1}$ using standard sectoriality estimates of *A*. In the case of a quasi-uniform partition $\Phi (t,s,n,j) = A (e^{-t_{n,j}A} - e^{-(t-s)A} )$, where $t_{n,j} :=\tau _n +\dots +\tau _{j+1}$, and thus similar calculations give $\Vert \Phi (t,s,n,j)\Vert ^2_{\mathcal {L}(X_{1/2},X_0)}\lesssim [\tau _{\max }(n-j-1)^2]^{-1}$. Therefore Theorem 3.12 also holds for quasi-uniform partitions. Theorem 3.13 follows from estimate (3.11), a consequence of Lemma 2.3. Since Lemmas 2.2 and 2.3 extend to quasi-uniform partitions (c.f. [58] and [51]), Theorem 3.13 also holds for quasi-uniform partitions. Finally note that Proposition 3.15 also extends to quasi-uniform partitions.

As a consequence, we obtain the following sufficient conditions for discrete stochastic maximal $\ell ^p$-regularity. In Hilbert spaces, we always have discrete maximal $\ell ^p$-regularity without any further conditions on *A* besides sectoriality of angle $<\pi /2$.

#### Corollary 3.8

($\textrm{DSMR}$ for free on Hilbert spaces). Suppose that Assumption 1.1 holds with $X_0$ a Hilbert space. Then *R* has discrete maximal $\ell ^p$-regularity on (0, *T*) for all $p\in [2, \infty )$ and $T\in (0,\infty ]$.

#### Proof

By the proof below [4, Theorem 3.13], *A* has $\textrm{SMR}(2,T)$. Therefore, arguing as in [41, Theorem 8.2] (see also [4, Theorem 3.12]), one obtains that *A* has $\textrm{SMR}(p,T)$ for any $p\in [2, \infty )$. The required result now follows from Theorem 3.6. $\square$

In $L^q$-spaces, the result holds under the additional condition that *A* has a bounded $H^\infty$-calculus of angle $<\pi /2$, which can be seen as a discrete analogue of the stochastic maximal regularity result of [46].

#### Corollary 3.9

($\textrm{DSMR}$ on $L^q$-spaces). Suppose that Assumption 1.1 holds and $X_0$ is isomorphic to a closed subspace of $L^q(\mathcal {O})$ with $q\in (2, \infty )$. Suppose that *A* has a bounded $H^\infty$-calculus of angle $<\pi /2$. Then *R* has discrete maximal $\ell ^p$-regularity on (0, *T*) for all $p\in (2, \infty )$ and $T\in (0,\infty ]$.

#### Proof

By [46, Theorem 1.1], *A* has $\textrm{SMR}(p,T)$ and thus *R* has $\textrm{DSMR}(p,T)$ by Theorem 3.6. $\square$

#### Remark 3.10

In the special case of the implicit Euler scheme $r(z) = (1+z)^{-1}$ and $0\in \rho (A)$, Corollary 3.9 follows from the work of [37]. In the latter paper, the regularity is shifted by a regularity of 1/2. However, due to BIP this leads to an equivalent definition as explained in Remark 3.3. The proof in [37] can be seen as a discrete analogue of the technique in [46]. Due to Theorem 3.6, one can directly apply the continuous-time setting and cover many more schemes at the same time.

On real interpolation spaces, the only additional condition needed is invertibility of *A*.

#### Corollary 3.11

($\textrm{DSMR}$ for free on real interpolation spaces). Let *A* be a sectorial operator of angle $<\pi /2$ on a space *E* with UMD and type 2, and suppose that $0\in \rho (A)$. Let $D_A(\alpha ,q) :=(E,D(A^n))_{\alpha /n,q}$ where $0<\alpha <n$ and $q\in [1, \infty ]$. Let $X_i :=D_A(\alpha +i,q), \ i\in \{0,1\},$ for some $\alpha >0$ and $q\in [2, \infty )$. Suppose that Assumption 1.1(2) holds. Then *R* has discrete maximal $\ell ^p$-regularity on (0, *T*) for all $p\in (2, \infty )$ and $T\in (0,\infty ]$.

#### Proof

It is well-known that *A* defines a sectorial operator of angle $<\pi /2$ on $X_0 = D_A(\alpha ,q)$. Moreover, *A* has a bounded $H^\infty$-calculus by Dore’s theorem (see [27, Corollary 16.3.22]). Thus, from Lemma 2.1 and the complex reiteration theorem (see [55, Section 1.10.3]) we obtain $D(A^{\theta }) = [D_A(\alpha ,q), D_A(\alpha +1,q)]_{\theta } = D_A(\alpha +\theta ,q)$ for all $\theta \in [0,1]$. Therefore, our definition of $\textrm{SMR}(p,T)$ coincides with the one used in [41, Theorem 8.6], and hence *A* has $\textrm{SMR}(p,T)$. It remains to apply Theorem 3.6. $\square$

### $\textrm{SMR}$ implies discrete $\textrm{SMR}$ for exponential Euler

As a first step, we prove that the exponential Euler method has discrete stochastic maximal regularity by a perturbation argument from the continuous-time setting.

#### Theorem 3.12

Suppose that Assumption 1.1 holds. Let $T\in (0,\infty ]$ and $p \in [2,\infty )$. If *A* has stochastic maximal $L^p$-regularity on (0, *T*), then the exponential Euler scheme (*EE*) has discrete maximal $\ell ^p$-regularity. Moreover,

$$\begin{aligned} C^{(EE)}_{\textrm{DSMR}(p,T)} \lesssim _{p,X_{0},A} C^A_{\textrm{SMR}(p,T)}+1, \end{aligned}$$

where the implicit constant only depends on *p*, *X*, and the sectoriality constant and angle of *A*.

#### Proof

Let $T\in (0,\infty ]$ and for admissible $\tau >0$ let $\pi _\tau :=\{t_n= n \tau :0 \le n \le N\}$ be a uniform partition of [0, *T*]. Let $g\in L^p_{\mathbb {F}}(\Omega ; L^p(0,T;\gamma (H,X_{1/2})))$. In order to shorten the notation, we write $\Vert g\Vert _{\Gamma _p}$ for the latter $L^p$-norm.

Since *A* has $\textrm{SMR}(p,T)$, we infer that

3.8 $$\begin{aligned} \left( \sum _{n=0}^{N-1} \int _{t_{n}}^{t_{n+1}} \mathbb {E}\Big \Vert A \int _0^t e^{-(t-s)A}g(s) \, dW(s) \Big \Vert _{X_0}^p dt \right) ^{1/p} \le C^A_{\textrm{SMR}(p,T)} \Vert g\Vert _{\Gamma _p}. \end{aligned}$$

In order to show that the exponential Euler (EE) scheme has $\textrm{DSMR}(p,T)$, it suffices to establish the estimate

$$\begin{aligned} \left( \sum _{n=1}^{N-1} \tau \, \mathbb {E}\Big \Vert A \sum _{j=0}^{n-1} e^{-\tau (n-j) A} \Delta _j I_g \Big \Vert _{X_0}^p \right) ^{1/p} \le C \Vert g\Vert _{\Gamma _p}. \end{aligned}$$

Note that by Proposition 2.5 and (2.4) we can estimate the $j=n-1$ term by

$$\begin{aligned} \left( \sum _{n=1}^{N-1} \tau \, \mathbb {E}\Vert A e^{-\tau A} \Delta _{n-1} I_g \Vert _{X_0}^p \right) ^{1/p}&\lesssim _{p,X_0} \left( \sum _{n=1}^{N-1} \tau \, \mathbb {E}\Vert A e^{-\tau A} g \Vert ^p_{L^2((t_{n}, t_{n+1});\gamma (H,X_0))} \right) ^{1/p} \\  &\lesssim _{p,X_0} \left( \sum _{n=1}^{N-1} \tau ^{\frac{p}{2}} \, \mathbb {E}\Vert A e^{-\tau A} g \Vert ^p_{L^p((t_{n}, t_{n+1});\gamma (H,X_0))} \right) ^{1/p} \\  &\lesssim _{p,X_0,A} \left( \sum _{n=1}^{N-1} \mathbb {E}\Vert g\Vert ^p_{L^p((t_{n}, t_{n+1});\gamma (H,X_{1/2}))} \right) ^{1/p} \\  &= \Vert g\Vert _{\Gamma _p}. \end{aligned}$$

Therefore it remains to prove that

3.9 $$\begin{aligned} \left( \sum _{n=2}^{N-1} \tau \, \mathbb {E}\Big \Vert A \sum _{j=0}^{n-2} e^{-\tau (n-j) A} \Delta _j I_g \Big \Vert _{X_0}^p \right) ^{1/p} \le C \Vert g\Vert _{\Gamma _p}. \end{aligned}$$

By the triangle inequality and (3.8), it suffices to establish the estimate

$$\begin{aligned}&\left( \sum _{n=2}^{N-1} \int _{t_{n}}^{t_{n+1}} \mathbb {E}\Big \Vert A \sum _{j=0}^{n-2} e^{-\tau (n-j) A} \Delta _j I_g - A \int _0^t e^{-(t-s)A}g(s) \, dW(s) \Big \Vert _{X_0}^p \, dt \right) ^{1/p}\\&\quad \le C \Vert g\Vert _{\Gamma _p}. \end{aligned}$$

To this end, let $2\le n\le N-1$ and $t \in [t_{n},t_{n+1}]$. Note that for $j \le n-1$ we have that $\textbf{1}_{[t_j,t_{j+1} \wedge t)} =\textbf{1}_{[t_j,t_{j+1})}$, whereas $\textbf{1}_{[t_j,t_{j+1} \wedge t)}=0$ for $j \ge n+1$ and so by the triangle inequality

$$\begin{aligned}&\left( \sum _{n=2}^{N-1} \int _{t_{n}}^{t_{n+1}} \mathbb {E}\Big \Vert A \sum _{j=0}^{n-2} e^{-\tau (n-j) A} \Delta _j I_g - A \int _0^t e^{-(t-s)A}g(s) \, dW(s) \Big \Vert _{X_0}^p dt\right) ^{1/p} \\&\le \left( \sum _{n=2}^{N-1} \int _{t_{n}}^{t_{n+1}} \mathbb {E}\Big \Vert \sum _{j=0}^{n-2} \int _{t_j}^{t_{j+1}}\Phi (t,s,n,j) g(s) dW(s) \Big \Vert _{X_0}^p dt\right) ^{1/p} \\  &\qquad + \left( \sum _{n=2}^{N-1} \int _{t_{n}}^{t_{n+1}}\mathbb {E}\Big \Vert \int _{t_{n-1}}^{t_n} A e^{-(t-s)} g(s)dW(s)\Big \Vert _{X_0}^p dt\right) ^{1/p} \\  &\qquad + \left( \sum _{n=2}^{N-1} \int _{t_{n}}^{t_{n+1}} \mathbb {E}\int _{t_n}^t Ae^{-(t-s)A} g(s) dW(s) \Big \Vert _{X_0}^p dt\right) ^{1/p}, \end{aligned}$$

where $\Phi (t,s,n,j):=A (e^{-\tau (n-j)A} - e^{-(t-s)A})$ appears in the first and main term.

The latter two terms can easily be estimated using the stochastic maximal regularity of *A*. Hence, it remains to prove the estimate

$$\begin{aligned} \sum _{n=2}^{N-1} \int _{t_{n}}^{t_{n+1}} \mathbb {E}\Big \Vert \int _0^T \Big ( \sum _{j=0}^{n-2} \Phi (t,s,n,j) g(s) \textbf{1}_{[t_j,t_{j+1})}(s) \Big ) dW(s) \Big \Vert ^p dt \le C^p \Vert g\Vert _{\Gamma _p}^p. \end{aligned}$$

By Proposition 2.5, it is enough to estimate

3.10 $$\begin{aligned} \sum _{n=2}^{N-1} \int _{t_{n}}^{t_{n+1}} \mathbb {E}\left( \sum _{j=0}^{n-2} \int _{t_j}^{t_{j+1}} \Vert \Phi (t,s,n,j) g(s)\Vert ^2_{\gamma (H,X_0)}\, ds \right) ^{p/2} dt \le C^p \Vert g\Vert _{\Gamma _p}^p. \end{aligned}$$

In order to estimate the latter, we first bound the operator norm of $\Phi (t,s,n,j)$. We claim that for $2\le n\le N-1$, $0\le j\le n-2$, $t_{n}\le t\le t_{n+1}$, and $t_j\le s\le t_{j+1}$

$$\begin{aligned} \Vert \Phi (t,s,n,j)\Vert _{\mathcal {L}(X_{1/2},X_0)}&\le \Vert \Phi (t,s,n,j)\Vert _{\mathcal {L}(X_{1},X_0)}^{1/2} \Vert \Phi (t,s,n,j)\Vert ^{1/2}_{\mathcal {L}(X_0)}\\&\lesssim \frac{1}{\tau ^{1/2} (n-j-1)}. \end{aligned}$$

Indeed, the first bound follows by interpolation and the $\Vert \Phi (t,s,n,j)\Vert _{\mathcal {L}(X_{1},X_0)}$-norm is clearly uniformly bounded. To estimate $\Vert \Phi (t,s,n,j)\Vert _{\mathcal {L}(X_0)}$, let $I_{nj}$ denote the interval with endpoints $t-s$ and $\tau (n-j)$. One can check that $|I_{nj}|\le \tau$ and $\min I_{nj} \ge \tau (n-j-1)$. Therefore, we write

$$\begin{aligned} \Vert \Phi (t,s,n,j)\Vert _{\mathcal {L}(X_0)} \le \int _{I_{nj}} \Vert A^2e^{-rA} \Vert dr \lesssim \frac{|I_{nj}|}{(\min I_{nj})^2}\le \frac{1 }{\tau (n-j-1)^2}, \end{aligned}$$

which clearly implies the claim. From the claim we obtain

$$\begin{aligned} \int _{t_j}^{t_{j+1}} \Vert \Phi (t,s,n,j) g(s)\Vert ^2_{\gamma (H,X_0)}\, ds&\lesssim \frac{1}{\tau (n-j-1)^{2}}\Vert g\Vert _{L^2(t_j, t_{j+1};\gamma (H,X_{1/2}))}^2. \end{aligned}$$

Using that the latter is *t*-independent, we see that the left-hand side of (3.10) is, up to a multiplicative constant, bounded by

$$\begin{aligned}&\tau ^{1-\frac{p}{2}} \mathbb {E}\sum _{n=2}^{N-1} \Big ( \sum _{j=0}^{{n}-2} { \textbf{1}_{n-j-1\ge 1} } \frac{1}{(n-j-1)^{2}} \Vert g\Vert _{L^2(t_j, t_{j+1};\gamma (H,X_{1/2}))}^2 \Big )^{p/2} \\&\qquad {\mathop {\lesssim }\limits ^{\text {(i)}}} \tau ^{1-\frac{p}{2}} \mathbb {E}\sum _{n=0}^{N-2} \Vert g\Vert _{L^2(t_n, t_{n+1};\gamma (H,X_{1/2}))}^p {\mathop {\le }\limits ^{\text {(ii)}}} \mathbb {E}\sum _{{n=1}}^{N-2} \Vert g\Vert _{L^p(t_n, t_{n+1};\gamma (H,X_{1/2}))}^p \\  &\qquad \le \mathbb {E}\Vert g\Vert _{L^p(0,T;\gamma (H,X_{1/2}))}^p, \end{aligned}$$

where we used the discrete case of Minkowski’s convolution inequality in (i) and Hölder’s inequality in (ii). $\square$

### Equivalence of discrete $\textrm{SMR}$ for different schemes

Our next step is to compare rational schemes to the exponential Euler scheme.

#### Theorem 3.13

Suppose that Assumption 1.1 holds, where $R_\tau :=r(\tau A)$ is the rational scheme introduced there. Let $T\in (0,\infty ]$ and $p \in (2,\infty )$. Then, the exponential Euler scheme has discrete maximal $\ell ^p$-regularity if and only if *R* has discrete maximal $\ell ^p$-regularity. Moreover,

$$\begin{aligned} C^{(EE)}_{\textrm{DSMR}(p,T)} +1 \eqsim _{p,X_0,r,A} C^{R}_{\textrm{DSMR}(p,T)}+1, \end{aligned}$$

where the implicit constant only depends on *p*, $X_0$, the rational function *r*, and the sectoriality constant and angle of *A*.

#### Proof of Theorem 3.13

By Lemma 2.3 we get that $\Vert A (e^{-n \tau A} - r(\tau A)^n)\Vert _{\mathcal {L}(X_0)}\lesssim _{r,A} \tau ^{-1} n^{-\ell -1}$ and $\Vert A (e^{-n \tau A} - r(\tau A)^n)\Vert _{\mathcal {L}(X_1, X_0)} \lesssim _{r,A} n^{-\ell }$. Hence by interpolation,

3.11 $$\begin{aligned} \Vert A (e^{-n \tau A} - r(\tau A)^n)\Vert _{\mathcal {L}(X_{1/2},X_0)} \lesssim _{r,A} \frac{1}{\tau ^{1/2}n^{\ell +1/2}}. \end{aligned}$$

Let $\tau >0$ be admissible, $\pi _\tau :=\{t_n = n \tau :0\le n \le N\}$ be a uniform partition of [0, *T*] and let $g \in L^p_{\mathbb {F}}(\Omega ;L^p(0,T;(\gamma (H,X_{1/2}))))$. To prove the equivalence, by the triangle inequality, it suffices to bound the difference of the schemes uniformly in $\tau$. Thus it suffices to establish the estimate

3.12 $$\begin{aligned} \sum _{n=1}^{N-1} \tau \, \mathbb {E}\Big \Vert \sum _{j=0}^{n-1}A \big ( e^{-\tau (n-j) A} -r(\tau A)^{n-j} \big ) \Delta _j I_g \Big \Vert _{X_0}^p \le C^p \Vert g\Vert _{L^p(\Omega ;L^p(0,T;(\gamma (H,X_{1/2}))))}^p . \end{aligned}$$

By Proposition 2.5 and (3.11), we can estimate

$$\begin{aligned}&\mathbb {E}\Big \Vert \sum _{j=0}^{n-1}A \big ( e^{-\tau (n-j) A} -r(\tau A)^{n-j} \big ) \Delta _j I_g \Big \Vert _{X_0}^p \\&\lesssim _{p,X_0} \mathbb {E}\left( \sum _{j=0}^{n-1}\int _{t_j}^{t_{j+1}} \, \Vert A \big ( e^{-\tau (n-j) A} -r(\tau A)^{n-j} \big ) g(s) \Vert ^2_{\gamma (H,X_0)} \, ds\right) ^{p/2} \\&\le \mathbb {E}\left( \sum _{j=0}^{n-1} \Vert A \big ( e^{-\tau (n-j) A} -r(\tau A)^{n-j} \big ) \Vert _{\mathcal {L}(X_{1/2},X_0)}^2 \, \Vert g\Vert ^2_{L^2(t_j, t_{j+1};\gamma (H,X_{1/2}))}\right) ^{p/2} \\&\lesssim _{r,A} \mathbb {E}\left( \sum _{j=0}^{n-1} \frac{1}{\tau (n-j)^{2\ell +1}} \, \Vert g\Vert ^2_{L^2(t_j, t_{j+1};\gamma (H,X_{1/2}))}\right) ^{p/2}. \end{aligned}$$

Therefore, up to a multiplicative constant, the left-hand side of (3.12) can be estimated by

$$\begin{aligned}&\sum _{n=1}^{N-1} \tau \, \mathbb {E}\left( \sum _{j=0}^{n-1} \frac{1}{\tau (n-j)^{2\ell +1}} \, \Vert g\Vert ^2_{L^2(t_j, t_{j+1};\gamma (H,X_{1/2}))}\right) ^{p/2}\\&\quad {\mathop {\lesssim }\limits ^{\text {(i)}}} \tau ^{1-\frac{p}{2}}\mathbb {E}\sum _{n=0}^{N-1} \Vert g\Vert ^{p}_{L^2(t_n, t_{n+1};\gamma (H,X_{1/2}))} \\&\quad {\mathop {\le }\limits ^{\text {(ii)}}} \mathbb {E}\sum _{n=0}^{N-1} \Vert g\Vert ^{p}_{L^p(t_n, t_{n+1};\gamma (H,X_{1/2}))} \\  &\quad \le \mathbb {E}\Vert g\Vert ^{p}_{L^p(0,T;\gamma (H,X_{1/2}))}, \end{aligned}$$

where we used the discrete case of Minkowski’s convolution inequality in (i) and Hölder’s inequality in (ii). $\square$

As a consequence, we immediately obtain the following:

#### Corollary 3.14

(Equivalence of $\textrm{DSMR}$ for all rational schemes). Suppose that Assumption 1.1(1) holds. Let $T\in (0,\infty ]$ and $p \in [2,\infty )$. Let *r*(*z*) and *s*(*z*) be consistent of order $\ge 1$, and $A(\theta )$-stable rational functions with $\theta \in (\omega (A), \frac{\pi }{2}]$ and $r(\infty )=s(\infty )=0$. Let $R_{\tau } = r(\tau A)$ and $S_{\tau } = s(\tau A)$. Then, the scheme *R* has discrete stochastic maximal $\ell ^p$-regularity on (0, *T*) if and only if *S* has discrete stochastic maximal $\ell ^p$-regularity on (0, *T*).

### Discrete SMR implies SMR

To prove that discrete stochastic maximal regularity implies its continuous analogue, we need a result on the convergence of the underlying scheme. The literature contains many results of this type, and the reader is, for instance, referred to [21, 28, 40] and references therein for an overview on this topic. The main feature of the result below is that the convergence rate seems to be optimal and contains parameters $\alpha$ and $\beta$, which entail parabolic smoothing. Even the choice $\alpha =\beta =0$ seems to be new in the setting we consider.

#### Proposition 3.15

Suppose that Assumption 1.1 holds. Let $0\le \alpha \le \beta \le 1$ be such that $\beta -\alpha <1/2$. Let $T \in (0,\infty ]$ and $p \in [2,\infty )$. For admissible $\tau$, let $\pi _\tau :=\{t_n = n\tau :0 \le n \le N \}$ be a uniform partition of [0, *T*] with $N = T/\tau$. Then there is a constant *C* depending only on $\alpha , \beta ,p,X_0,X_1$, the sectoriality constants of *A*, and the function *r*, such that for every piecewise constant $g :=\sum _{n=0}^{N-1} g_n \textbf{1}_{[t_{n},t_{n+1})}$ with $g_n \in L^p(\Omega ,{\mathscr {F}}_{t_n},\mathbb {P}; \gamma (H,X_{\alpha }))$, $0 \le n \le N-1$,

$$\begin{aligned} \Big (\sum _{n=0}^{N-1} \Vert A^{\beta }(y-Y_n)\Vert ^p_{L^p((t_n,t_{n+1})\times \Omega ; X_0)} \Big )^{1/p} \le C \tau ^{\frac{1}{2}+\alpha -\beta } \Vert g\Vert _{L^p((0,T)\times \Omega ; \gamma (H,X_\alpha ))}, \end{aligned}$$

where *Y* is the discrete solution defined in (3.1) and *y* is the mild solution as defined in (3.5).

The constant *C* does not depend on $\tau$ and *T*. The above result can be seen as an optimal convergence result with parabolic smoothing.

#### Proof

Let $n\ge 0$ and $t\in (t_n, t_{n+1}]$. We split the expression as

$$\begin{aligned}&\Vert A^{\beta }(y(t)-Y_n)\Vert _{X_0} \\&\quad =\Big \Vert A^{\beta } \int _0^t e^{-(t-s)A} g(s) \,\textrm{d}W(s)- \sum _{j=0}^{n-1} A^{\beta } R_\tau ^{n-j} \Delta _j I_g \Big \Vert _{X_0} \\&\quad = \Big \Vert \sum _{j=0}^{n-1} \int _{t_j}^{t_{j+1}} A^{\beta }(e^{-(t-s)A} - R_\tau ^{n-j}) g_j \,\textrm{d}W(s)+ \int _{t_n}^t A^\beta e^{-(t-s)A} g_n \,\textrm{d}W(s)\Big \Vert _{X_0} \\&\quad \le \Big \Vert \sum _{j=0}^{n-1} \int _{t_j}^{t_{j+1}} A^{\beta }(e^{-(t-s)A} - e^{-\tau (n-j)A} ) g_j \,\textrm{d}W(s)\Big \Vert _{X_0} \\  &\quad + \Big \Vert \sum _{j=0}^{n-1} \int _{t_j}^{t_{j+1}} A^{\beta }(e^{-\tau (n-j)A} -R_\tau ^{n-j}) g_j \,\textrm{d}W(s)\Big \Vert _{X_0} + \Big \Vert \int _{t_n}^t A^{\beta }e^{-(t-s)A} g_n \,\textrm{d}W(s)\Big \Vert _{X_0} \\&\quad :=I_{1,n}(t)+I_{2,n}(t) + I_{3,n}(t), \end{aligned}$$

where we set $I_{1,0} = I_{2,0} = 0$.

We estimate the $L^p(\Omega )$ norms of $I_{1,n}(t), I_{2,n}(t)$ and $I_{3,n}(t)$ as follows. For $I_{3,n}(t)$, Proposition 2.5 gives that

$$\begin{aligned} \Vert I_{3,n}(t)\Vert ^p_{L^p(\Omega )}&\lesssim \mathbb {E}\Big ( \int _{t_n}^t \Vert A^{\beta }e^{-(t-s)A}\Vert ^2_{\mathcal {L}(X_\alpha ,X_0)} \Vert g_n\Vert _{\gamma (H,X_\alpha )}^2 ds \Big )^{p/2} \\  &\lesssim \mathbb {E}\Big ( \int _{t_n}^t (t-s)^{-2(\beta -\alpha )}\Vert g_n\Vert _{\gamma (H,X_\alpha )}^2 ds \Big )^{p/2} \\  &\lesssim \tau ^{p (\frac{1}{2}-(\beta -\alpha ))} \mathbb {E}\Vert g_n\Vert ^p_{\gamma (H,X_\alpha )}. \end{aligned}$$

To estimate $I_{1,n}(t)$, note that by Proposition 2.5,

3.13 $$\begin{aligned} \Vert I_{1,n}(t)\Vert ^p_{L^p(\Omega )}&\lesssim \mathbb {E}\Big ( \sum _{j=0}^{n-1} \int _{t_j}^{t_{j+1}} \Vert A^{\beta }(e^{-(t-s)A} - e^{-\tau (n-j)A}) g_j\Vert ^2_{\gamma (H,X_0)} \, ds \Big )^{p/2} \nonumber \\&\le \mathbb {E}\Big ( \sum _{j=0}^{n-1} \int _{t_j}^{t_{j+1}} \Vert A^{\beta }(e^{-(t-s)A} - e^{-\tau (n-j)A}) \Vert _{\mathcal {L}(X_\alpha ,X_0)}^2 \, \Vert g_j\Vert ^2_{\gamma (H,X_\alpha )} \, ds \Big )^{p/2}.\nonumber \\ \end{aligned}$$

For the term $j=n-1$ and $s\in (t_{n-1}, t_{n})$ we can estimate

$$\begin{aligned} \Vert A^{\beta }(e^{-(t-s)A} - e^{-\tau A})\Vert _{\mathcal {L}(X_\alpha ,X_0)}&\le \Vert A^{\beta } e^{-(t-s)A}\Vert _{\mathcal {L}(X_\alpha ,X_0)} + \Vert A^{\beta }e^{-\tau A} \Vert _{\mathcal {L}(X_\alpha ,X_0)} \\&\lesssim (t-s)^{-(\beta -\alpha )}. \end{aligned}$$

Therefore,

$$\begin{aligned}&\int _{t_{n-1}}^{t_{n}} \Vert A^{\beta }( e^{-(t-s)A} - e^{-\tau A} )\Vert _{\mathcal {L}(X_\alpha ,X_0)}^2 \, \Vert g_{n-1}\Vert ^2_{\gamma (H,X_\alpha )} \, ds \\&\quad \lesssim \int _{t_{n-1}}^{t_n} (t-s)^{-2(\beta -\alpha )} \Vert g_{n-1}\Vert ^2_{\gamma (H,X_\alpha )} ds \\  &\quad \lesssim \tau ^{1-2(\beta -\alpha )} \Vert g_{n-1}\Vert ^2_{\gamma (H,X_\alpha )} \end{aligned}$$

and thus

$$\begin{aligned}&\mathbb {E}\Big (\int _{t_{n-1}}^{t_{n}} \Vert A^{\beta }(e^{-(t-s)A} - e^{-\tau A}) \Vert _{\mathcal {L}(X_\alpha ,X_0)}^2 \, \Vert g_{n-1}\Vert ^2_{\gamma (H,X_\alpha )} \, ds\Big )^{p/2}\\&\quad \lesssim \tau ^{p (\frac{1}{2}-(\beta -\alpha ))} \mathbb {E}\Vert g_{n-1}\Vert ^p_{\gamma (H,X_\alpha )}. \end{aligned}$$

For $j\le n-2$ and $s\in (t_j, t_{j+1}]$, by (2.5) we can estimate

$$\begin{aligned} \Vert&A^{\beta }(e^{-(t-s)A} - e^{-\tau (n-j)A}) \Vert _{\mathcal {L}(X_{\alpha },X_0)} \\  &\le \Vert A^{\beta -1} (e^{-(t-s - (n-j-1)\tau )A} - e^{-\tau A})\Vert _{\mathcal {L}(X_\beta , X_0)} \Vert A e^{-(n-j-1)\tau A}\Vert _{\mathcal {L}(X_\alpha ,X_\beta )} \\  &\lesssim (t-s-\tau (n-j)) ((n-j-1)\tau )^{-(1+\beta -\alpha )} \le \tau ^{-(\beta -\alpha )} (n-j-1)^{-(1+\beta -\alpha )}. \end{aligned}$$

Substituting this into (3.13) gives

$$\begin{aligned}&\Vert I_{1,n}(t)\Vert ^p_{L^p(\Omega )} \\&\quad \lesssim \tau ^{p (\frac{1}{2}-(\beta -\alpha ))} \Big [ \mathbb {E}\Big ( \sum _{j=0}^{n-2} \frac{1}{(n-j-1)^{2(1+\beta -\alpha )}}\Vert g_j\Vert ^2_{\gamma (H,X_{\alpha })} \Big )^{p/2} + \mathbb {E}\Vert g_{n-1}\Vert ^p_{\gamma (H,X_\alpha )}\Big ]. \end{aligned}$$

We now estimate $I_{2,n}(t)$. By Proposition 2.5,

3.14 $$\begin{aligned} \Vert I_{2,n}(t)\Vert ^p_{L^p(\Omega )}&\lesssim \mathbb {E}\Big ( \sum _{j=0}^{n-1} \int _{t_j}^{t_{j+1}} \Vert A^\beta (e^{-\tau (n-j)A} -R_\tau ^{n-j}) g_j\Vert ^2_{\gamma (H,X_0)} \, ds \Big )^{p/2} \nonumber \\&\le \mathbb {E}\Big ( \sum _{j=0}^{n-1} \int _{t_j}^{t_{j+1}} \Vert A^\beta ( e^{-\tau (n-j)A} -R_\tau ^{n-j})\Vert _{\mathcal {L}(X_{\alpha },X_0)}^2 \, \Vert g_j\Vert ^2_{\gamma (H,X_\alpha )} \, ds \Big )^{p/2} . \end{aligned}$$

By (2.12), we get that $\Vert A^{\beta } (e^{-\tau (n-j)A} - R_\tau ^{n-j})\Vert _{\mathcal {L}(X_\alpha ,X_0)} \lesssim \tau ^{-(\beta -\alpha )} (n-j)^{-1-(\beta -\alpha )}$ and thus (3.14) gives that

$$\begin{aligned} \Vert I_{2,n}(t)\Vert ^p_{L^p(\Omega )} \lesssim \tau ^{p(\frac{1}{2}-(\beta -\alpha ))} \mathbb {E}\Big ( \sum _{j=0}^{n-1} \frac{1}{(n-j)^{2(1+\beta -\alpha )}} \Vert g_j\Vert ^2_{\gamma (H,X_\alpha )} \, ds \Big )^{p/2} . \end{aligned}$$

Finally, applying the discrete case of Minkowski’s convolution inequality, we can conclude that

$$\begin{aligned} \sum _{n=1}^{N-1} \Vert A^\beta (y-Y_n)\Vert ^p_{L^p((t_n,t_{n+1})\times \Omega ;X_0)}&\le \sum _{k=1}^3 \sum _{n=0}^{N-1} \int _{t_n}^{t_{n+1}} \Vert I_{k,n}(t)\Vert _{L^p(\Omega )}^p dt \\  &\lesssim \tau ^{p(\frac{1}{2}-(\beta -\alpha ))} \mathbb {E}\sum _{n=0}^{N-1} \tau \Vert g_n\Vert ^p_{\gamma (H,X_\alpha )} . \end{aligned}$$

$$\square $$

#### Remark 3.16

Proposition 3.15 seems to be new in this generality. Under the assumption that *A* has deterministic maximal $L^p$-regularity and $0\in \rho (A)$, a similar result was shown in [37, Theorem 4.1] for $\alpha =\beta =0$ and for the implicit Euler scheme with an entirely different method.

#### Theorem 3.17

Suppose that Assumption 1.1 holds. Let $T\in (0,\infty ]$ and $p \in [2,\infty )$. If *R* has discrete stochastic $\ell ^p$-maximal regularity on (0, *T*), then *A* has stochastic maximal $L^p$-regularity on (0, *T*).

#### Proof

Let $\tau _0>0$ be admissible and fixed and let $\pi _{\tau _0} :=\{t_n^0=n \tau _0:\ 0\le n \le N_0 \}$ be a uniform partition of [0, *T*]. Let $g :=\sum _{n=0}^{N-1} g_n \textbf{1}_{[t_n^0,t_{n+1}^0)}$ be fixed but arbitrary, where each $g_n \in L^p(\Omega ,{\mathscr {F}}_{t_n},\mathbb {P}; \gamma (H,D(A))$ and let $y(t):=\int _0^t e^{-(t-s)A} g(s) \,\textrm{d}W(s)$. Since the set of such processes *g* is dense in $L^p_{\mathbb {F}} ((0,T)\times \Omega ; \gamma (H,X_{1/2}))$, it suffices to show that

3.15 $$\begin{aligned} \Vert A y \Vert _{L^p((0,T)\times \Omega ;X_0)} \le C \Vert g\Vert _{L^p((0,T)\times \Omega ;\gamma (H,X_{1/2}))}. \end{aligned}$$

For each integer $k \ge 1$ let $\tau _k= \tau _0/k$ and let $\pi _{\tau _k} :=\{t_n^k = n \tau _k :0 \le n \le N_k\}$ with $N_k = T/\tau$ if $T<\infty$ and $N_k=\infty$ otherwise. We can write $g= \sum _{n=0}^{N_k-1} g_n^k \textbf{1}_{[t_n^k,t_{n+1}^k)}$, where each $g_n^k \in L^p(\Omega ,{\mathscr {F}}_{t_n^k},\mathbb {P}; \gamma (H,X_1))$ and let $Y_n^k :=\sum _{j=0}^{n-1} R_{\tau _k}^{n-j} \Delta _j I_{g}$, where $\Delta _j I_{g} :=I_g(t_{j+1}^k) - I_g(t_{j}^k)$. Proposition 3.15 gives that

$$\begin{aligned} \Big ( \sum _{n=0}^{N_k-1} \int _{t_n}^{t_{n+1}} \mathbb {E}\Vert A Y_n^k - A y(t) \Vert ^p_{X_0} dt \Big )^{1/p} \le C \tau _k^{\frac{1}{2}} \Vert g\Vert _{L^p((0,T)\times \Omega ;\gamma (H,X_1))} \end{aligned}$$

and since *R* has $\textrm{DSMR}(p,T)$,

$$\begin{aligned} \Big ( \sum _{n=0}^{N_k-1} \tau \mathbb {E}\Vert A Y_n^k\Vert ^p_{X_0} \Big )^{1/p} \le C_{\text {DSMR}(p,T)}^R \Vert g\Vert _{L^p((0,T)\times \Omega ;\gamma (H,X_{1/2}))}. \end{aligned}$$

Hence, by the triangle inequality,

$$\begin{aligned}&\Vert Ay \Vert _{L^p((0,T)\times \Omega ;X_0)} \\&\quad \le C \tau _k^{\frac{1}{2}} \Vert g\Vert _{L^p((0,T)\times \Omega ;\gamma (H,X_{1}))} + C_{\textrm{DSMR}(p,T)}^R \Vert g\Vert _{L^p((0,T)\times \Omega ;\gamma (H,X_{1/2}))}, \end{aligned}$$

and by letting $k \rightarrow \infty$, we obtain (3.15). This shows that *A* has stochastic $L^p$-maximal regularity on (0, *T*) with constant $C_{\text {SMR}(p,T)}^A \le C_{\textrm{DSMR}(p,T)}^R$. $\square$

Observe that Theorem 3.6 follows by combining Theorems 3.12, 3.13, and 3.17. In particular, this proves Theorem 1.2.

## Permanence properties

Before we move on to the proof of the maximal estimate of Theorem 1.4, we discuss some simple properties of discrete stochastic maximal $\ell ^p$-regularity, which we can now deduce from Theorem 3.6. Similar results have been discussed in the continuous-time setting in the deterministic case in [14] (also see [27, Section 17.2.e]), and in the stochastic case in [2, 41]. In Sect. 4.1, we recall some of the required permanence properties in continuous time, and in Sect. 4.2 derive its discrete analogues. Finally, in Sect. 4.3 we present a weighted extrapolation result for $\textrm{DSMR}$.

### The continuous-time setting

In the next result, we first collect some continuous-time results which can be found in [2, 41]. The definition in the latter two papers differs from the one we use here, since we shifted the smoothness by 1/2, and consider complex interpolation spaces instead of fractional powers. Therefore, we need to indicate the necessary changes in the proofs.

Note that Definition 3.5 does not require Assumption 1.1, but it is enough to assume that $-A$ generates a strongly continuous analytic semigroup.

#### Proposition 4.1

(Continuous-time setting). Let $-A$ generate a strongly continuous analytic semigroup on $X_0$. Let $p\in [2, \infty )$ and $T\in (0,\infty ]$. Suppose that *A* has stochastic maximal $L^p$-regularity on (0, *T*) with respect to a cylindrical Brownian motion on *H* with $\text {dim}(H)\ge 1$. Then the following hold:

1. If $T<\infty$ and $\lambda \in \mathbb {C}$, then $A+\lambda \in \textrm{SMR}(p,T)$;
2. If $T=\infty$ and $\Re (\lambda )\ge 0$, then $A+\lambda \in \textrm{SMR}(p,\infty )$;
3. If $T<\infty$ and $\lim _{t\rightarrow \infty }\Vert e^{-tA}\Vert _{\mathcal {L}(X_0)} = 0$, then $A\in \textrm{SMR}(p,\infty )$;
4. If $$\widetilde{T}\in (0,\infty )$$, then $$A\in \textrm{SMR}(p,\widetilde{T})$$;
5. If $$q\in (2, \infty )$$, then $$A\in \textrm{SMR}(q,T)$$;
6. If $\widetilde{H}$ is another Hilbert space, then $A\in \textrm{SMR}(p,T)$ with respect to any cylindrical Brownian motion on $\widetilde{H}$.

#### Proof

(1), (2): This can be proved in a similar way as [2, Proposition 3.8].

(3): The proof of [2, Theorem 5.2] extends to our setting.

(4): The same argument as in [2, Proposition 5.1 and Corollary 5.3] can be applied.

(5): The *p*-independence for $T = \infty$ can be proved as in [41, Theorem 8.2]. If $T<\infty$, then we can use a simple shift argument. Let $\lambda \ge 0$ be such that $\lim _{t\rightarrow \infty }\Vert e^{-t(\lambda +A)}\Vert _{\mathcal {L}(X_0)}=0$. By (2), $\lambda +A\in \textrm{SMR}(p,T)$, and thus $\lambda +A\in \textrm{SMR}(p,\infty )$ by (3). Hence $\lambda +A\in \textrm{SMR}(q,\infty )$. By (4) and (1) this implies $\lambda +A\in \textrm{SMR}(q,T)$.

(6): This can be proved in a similar way as in [2, Theorem 3.9]. $\square$

We do not know whether the assumption that $-A$ generates a strongly continuous analytic semigroup on $X_0$ can be weakened. Some results in this direction can be found in [2, Theorem 4.1].

### The discrete setting

The main result on permanence properties in the discrete case can be formulated as follows.

#### Theorem 4.2

Suppose that Assumption 1.1 holds. Let $p\in [2, \infty )$ and $T\in (0,\infty ]$. Suppose that *R* has discrete stochastic maximal $\ell ^p$-regularity on (0, *T*) with respect to a cylindrical Brownian motion on *H* with $\text {dim}(H)\ge 1$. Then the following hold:

1. If $\widetilde{T}\in (0,\infty )$, then *R* has discrete stochastic maximal $\ell ^p$-regularity on $(0,\widetilde{T})$;
2. If $T<\infty$ and $\lim _{t\rightarrow \infty }\Vert e^{-tA}\Vert _{\mathcal {L}(X_0)} = 0$, then *R* has discrete stochastic maximal $\ell ^p$-regularity on $(0,\infty )$;
3. If $q\in (2, \infty )$, then *R* has discrete stochastic maximal $\ell ^q$-regularity on (0, *T*);
4. If $\widetilde{H}$ is another Hilbert space, then *R* has discrete stochastic maximal $\ell ^p$-regularity on (0, *T*) with respect to any cylindrical Brownian motion on $\widetilde{H}$.

#### Proof

All properties are immediate from Proposition 4.1 and Theorem 3.6. $\square$

### Weighted extrapolation

In the previous result, we saw that discrete stochastic maximal regularity is independent of *p*, *T*, and *H*. Below, we will show that it is also equivalent to a weighted variant. In the continuous-time setting, such weighted results form a central tool in the theory of evolution equations (see [4, 50, 57]). The following can be seen as the discrete stochastic analogue of [2, Theorem 7.9] and [49, Theorem 2.4].

To extend Definition 3.1 to the weighted setting, let $p\in (2, \infty )$, $\alpha \in (-1,\frac{p}{2}-1)$ and set $w_{\alpha }(t) = t^{\alpha }$. We say that $R\in \textrm{DSMR}(p,\alpha ,T)$ in case Definition 3.1 is satisfied with the estimate (3.3) replaced by

$$\begin{aligned} \Vert A Y \Vert _{L^p(\Omega ;\ell ^p_{\tau ,w_{\alpha }}(X_{0}))} \le C \Vert g\Vert _{L^p(\Omega ;L^p (0,T,w_{\alpha };\gamma (H,X_{1/2})))}. \end{aligned}$$

Here, for $y = (y_n)_{n\ge 0}$ in $X_0$, we set

$$\begin{aligned} \Vert y\Vert _{\ell ^p_{\tau ,w_{\alpha }}(X_0)} = \left( \sum _{n\ge 0} \tau w_{\alpha }((n+1)\tau ) \Vert y_n\Vert _{X_0}^p\right) ^{1/p} = \left( \sum _{n\ge 0} \tau w_{\alpha }(t_{n+1}) \Vert y_n\Vert _{X_0}^p\right) ^{1/p}, \end{aligned}$$

and for $f:(0,T)\rightarrow Z$ we write

$$\begin{aligned} \Vert f\Vert _{L^p (0,T,w_{\alpha };Z)} = \Big (\int _{0}^T \Vert f(t)\Vert _Z^p w_{\alpha }(t) dt\Big )^{1/p}. \end{aligned}$$

In a similar way, one can define $A\in \textrm{SMR}(p,\alpha ,T)$ by including weights in Definition 3.5.

The following result is a weighted extension of Theorem 3.6.

#### Theorem 4.3

Suppose that Assumption 1.1 holds. Let $p\in (2, \infty )$, $\alpha \in (-1,\frac{p}{2}-1)$, and $T\in (0,\infty ]$. Then the following are equivalent:

1. *A* has $$\textrm{SMR}(p,T)$$;
2. *R* has $$\textrm{DSMR}(p,T)$$;
3. *A* has $$\textrm{SMR}(p,\alpha ,T)$$;
4. *R* has $$\textrm{DSMR}(p,\alpha ,T)$$.

This result will be derived from a more general extrapolation result for more general kernels.

#### Proposition 4.4

Let *Y* be a Banach space, and *Z* be a Banach space with UMD and type 2. Let $\Delta = \{(n,j) \in \mathbb {N}^2:0\le j<n\}$. Let $p\in (2, \infty )$ and $\alpha \in (-1, \frac{p}{2}-1)$. Suppose that $K:\Delta \rightarrow \mathcal {L}(Y,Z)$ is such that, for some constant $M\ge 0$,

$$\begin{aligned} \Vert K(n,j)\Vert _{\mathcal {L}(Y,Z)}\le \frac{M}{(\tau (n-j))^{1/2}}, \quad (n,j)\in \Delta . \end{aligned}$$

For an adapted step process $g:\mathbb {R}_+\times \Omega \rightarrow \gamma (H,Y)$, let $S_K g$ be the $L^p(\Omega ;Y)$-valued sequence given by

$$\begin{aligned} (S_K g)_0 :=0 \quad \text { and } \quad (S_K g)_n :=\sum _{j=0}^{n-1} K(n,j) \Delta _j I_g,\quad n \ge 1. \end{aligned}$$

Then the following are equivalent

1. $S_K$ extends to a bounded operator with
  $$\begin{aligned} \Vert S_K\Vert _{\mathcal {L}(L^p_{\mathbb {F}} (\Omega ; L^p (0,T;\gamma (H,Y))), L^p(\Omega ;\ell ^p_{\tau }(Z)))}\le C_1; \end{aligned}$$
2. $S_K$ extends to a bounded operator with
  $$\begin{aligned} \Vert S_K\Vert _{\mathcal {L}(L^p_{\mathbb {F}} (\Omega ; L^p (0,T,w_{\alpha };\gamma (H,Y))),L^p(\Omega ;\ell ^p_{\tau ,w_{\alpha }}(Z)))}\le C_2. \end{aligned}$$

Moreover, there is a constant $C_{\alpha ,p,Z}$ only depending on $\alpha , p, Z$ such that $C_1\le C_2 + C_{\alpha , p,Z} M$ and $C_2\le C_1 + C_{\alpha , p,Z} M$.

To prove this, we need the following.

#### Lemma 4.5

Suppose that the assumptions of Proposition 4.4 are satisfied. Let $\beta \in (-\infty , \frac{1}{2} - \frac{1}{p})$. Then $S_{K, \beta }:L^p_{\mathbb {F}} (\Omega ; L^p (0,T;\gamma (H,Y)))\rightarrow L^p(\Omega ;\ell ^p_{\tau }(Z))$ defined by

$$\begin{aligned}&(S_{K,\beta } g)_0 :=0 \quad \text { and } \\&(S_{K, \beta }g)_n :=\sum _{j=0}^{n-1} K(n,j) \int _{t_j}^{t_{j+1}} [(t_{n+1}/s)^{\beta }-1] g(s) d W(s), \quad n \ge 1, \end{aligned}$$

is bounded of norm $\Vert S_{K, \beta }\Vert \le C_{p,Z} C_{\beta } M$.

#### Proof

By Proposition 2.5

$$\begin{aligned} \mathbb {E}\Vert (S_{K, \beta }g)_n\Vert ^p_Z&\le C_{p,Z}^p \mathbb {E}\left( \sum _{j=0}^{n-1} \int _{t_j}^{t_{j+1}} \Vert K(n,j) [(t_{n+1}/s)^{\beta }-1] g(s)\Vert _{\gamma (H,Z)}^2 ds\right) ^{p/2} \\  &\le C_{p,Z}^p M^p \mathbb {E}\left( \sum _{j=0}^{n-1} \int _{t_j}^{t_{j+1}} \frac{1}{t_{n+1}-t_{j+1}}|(t_{n+1}/s)^{\beta }-1|^2 \Vert g(s)\Vert _{\gamma (H,Y)}^2 ds\right) ^{p/2}. \end{aligned}$$

It is elementary to check that for any $t\in (t_{n+1}, t_{n+2})$ and $s\in (t_j, t_{j+1})$,

4.1 $$\begin{aligned} \frac{1}{t_{n+1}-t_{j+1}}|(t_{n+1}/s)^{\beta }-1|^2\le \frac{3}{t-s}|(t/s)^{\beta }-1|^2. \end{aligned}$$

Therefore, since $$(S_{K, \beta }g)_0 = 0$$,

$$\begin{aligned} \sum _{n\ge 0} \tau \mathbb {E}\Vert (S_{K, \beta }g)_n\Vert ^p_Z&= \sum _{n\ge 1} \int _{t_{n+1}}^{t_{n+2}} \mathbb {E}\Vert (S_{K, \beta }g)_n\Vert ^p_Z dt \\  &\le 3^{p/2} (C_{p,Z})^p M^p \int _{0}^\infty \mathbb {E}\left( \int _{0}^{t} \frac{1}{t-s}|(t/s)^{\beta }-1|^2 \Vert g(s)\Vert _{\gamma (H,Y)}^2 ds\right) ^{p/2} dt \\  &\le 3^{p/2} (C_{p,Z})^p M^p C_{p,\beta }^p \Vert g\Vert _{L^p(\Omega ; L^p (0,T;\gamma (H,Y)))}^p, \end{aligned}$$

where in the last step we used an estimate of the proof of [2, Lemma 7.11]. $\square$

By the above lemma, the proof of the proposition follows from the identity

$$\begin{aligned} (S_K g)_n = t_{n+1}^{\beta } (S_K g_{-\beta })_n - (S_{K,\beta } g)_n, \end{aligned}$$

where $\beta = \alpha /p$, $g_{-\beta }(s) = s^{-\beta } g(s)$. Since the argument is almost identical to [2, Theorem 7.10], the details are left to the reader. $\square$

#### Proof of Theorem 4.3

(2) $\Leftrightarrow$ (4): It remains to observe that by (2.13) and an interpolation argument, the kernel $K(n,j):=A R_{\tau }^{n-j}$ satisfies $\Vert K(n,j)\Vert _{\mathcal {L}(X_{1/2}, X_0)}\le \frac{M}{(\tau (n-j))^{1/2}}$ for every $n>j \ge 0$.

(1) $\Leftrightarrow$ (3): This can be proved as in [2, Theorem 7.9] by shifting the space regularity by 1/2.

(1) $\Leftrightarrow$ (2): This is the content of Theorem 3.6. $\square$

#### Remark 4.6

(Quasi-uniform partitions). Theorem 4.3 extends to quasi-uniform partitions $\pi$ given by variable time-steps $(\tau _n)_{n=1}^N$ satisfying $\mu ^{-1} \le \frac{\tau _{\max }}{\tau _{\min }} \le \mu$. In this case the weighted norm is given by $\Vert y\Vert ^p_{\ell ^p_{\pi ,w_{\alpha }}(X_0)} =\sum _{n\ge 0} \tau _{n+1} w_{\alpha }(t_{n+1}) \Vert y_n\Vert _{X_0}^p$. Indeed, the unweighted equivalence (1) $\Leftrightarrow$ (2) is established in Remark 3.7, whereas the equivalence (1) $\Leftrightarrow$ (3) follows from the continuous-time setting ([2, Theorem 7.9]). In order to prove the equivalence (2) $\Leftrightarrow$ (4) it suffices to observe that the kernel estimate $\Vert AR_\pi ^{n,j}\Vert _{\mathcal {L}(X_{1/2}, X_0)}\le M(t_n-t_j)^{-1/2}$ holds (see Remark 3.7) and that the proofs of Proposition 4.4 and Lemma 4.5 extend mutatis mutandis to the quasi-uniform setting. The only significant modification is that the elementary estimate (4.1) of Lemma 4.5 requires the constant $C_\mu = 1+2\mu$ instead of 3.

## $\mathcal {R}$-boundedness of discrete stochastic convolutions

One of the ingredients in the proof of the maximal estimate in Theorem 1.4 is an $\mathcal {R}$-boundedness result for discrete stochastic convolutions. Here, the prefix $\mathcal {R}$ does not refer to the scheme, and therefore we have chosen to use a calligraphic letter instead. $\mathcal {R}$-boundedness plays a crucial role in vector-valued harmonic and stochastic analysis, and the letter $\mathcal {R}$ refers to Rademacher or random. For an overview of $\mathcal {R}$-boundedness and its role in analysis, the reader is referred to [26, Chapter 8].

### Definitions

Let $(r_n)_{n\ge 1}$ be a Rademacher sequence, i.e. $\mathbb {P}(r_n = +1) = \mathbb {P}(r_n=-1) = 1/2$, and the random variables $(r_n)_{n\ge 1}$ are independent. Let *Y* and *Z* be Banach spaces. A family of operators $\mathcal {T}\subseteq \mathcal {L}(Y,Z)$ is said to be $\mathcal {R}$-*bounded* if there exists a constant $C\ge 0$ such that for all finite sequences $(T_n)_{n=1}^N$ in $\mathcal {T}$ and $(y_n)_{n=1}^N$ in *Y*,

$$\begin{aligned} \Big \Vert \sum _{n=1}^N r_n T_n y_n\Big \Vert _{L^2(\Omega ;Z)} \le C\Big \Vert \sum _{n=1}^N r_n y_n\Big \Vert _{L^2(\Omega ;Y)}. \end{aligned}$$

The least admissible constant in the above estimate is called the $\mathcal {R}$-*bound* of $\mathcal {T}$ and is denoted by $\mathcal {R}(\mathcal {T})$.

### Main $\mathcal {R}$-boundedness result

In the continuous-time setting $\mathcal {R}$-boundedness of stochastic convolutions was obtained in [46, 47] in order to establish stochastic maximal $L^p$-regularity. These methods were extended to a discrete setting for implicit Euler in [37], to establish discrete stochastic maximal regularity.

Unless stated otherwise, in the rest of this section $X_0$ is assumed to be isomorphic to a closed subspace of $L^q(\mathcal {O})$ with $(\mathcal {O}, \Sigma ,\mu )$ a $\sigma$-finite measure space. Given a stepsize $\tau >0$, $\mathcal {K}_{\tau }$ denotes the set of all sequences $k = (k_n)_{n\ge 1}$ such that $k_n \rightarrow 0$ and

$$\begin{aligned} \sum _{n \ge 1} \sqrt{n \tau } \, |k_{n+1}-k_n| \le 1. \end{aligned}$$

For $k =(k_n)_{n\ge 1} \in \mathcal {K}_{\tau }$ and an elementary adapted process $g:[0,\infty ) \times \Omega \rightarrow L^q(\mathcal {O};H)$ we define the process $I^{\tau }(k)g :\mathbb {N}\times \Omega \rightarrow L^q(\mathcal {O})$ via

$$\begin{aligned} (I^{\tau }(k)g)_0 :=0 \quad \text {and } \quad (I^{\tau }(k)g)_n :=\sum _{j=0}^{n-1} k_{n-j} \Delta _j I_g, \quad n \ge 1, \end{aligned}$$

where we recall that $I_g(t) = \int _0^t g d W$ and $\Delta _j I_g = I_g(t_{j+1}) - I_g(t_{j})$. Finally, let $\mathcal {I}^{\tau } :=\{I^{\tau }(k):k\in \mathcal {K}_{\tau }\}$.

Recall that the notation for the function spaces with weights $w_{\alpha }(t) = t^{\alpha }$, was introduced in Sect. 4.3.

#### Theorem 5.1

Let $q\in [2, \infty )$. Let $p\in (2, \infty )$ and $\alpha \in (-1,\frac{p}{2}-1)$. In case $q=2$, we additionally allow $p=2$ and $\alpha =0$. Then there exists a constant $C_{p,q,\alpha }$ such that for all $\tau >0$ the family

$$\begin{aligned} \mathcal {I}^{\tau } \subseteq \mathcal {L}( L^p_{\mathbb {F}}(\Omega ;L^p(\mathbb {R}_+,w_{\alpha };L^q(\mathcal {O};H))), L^p(\Omega ; \ell ^p_{\tau ,w_{\alpha }}(L^q(\mathcal {O}))) ) \end{aligned}$$

is $\mathcal {R}$-bounded by $C_{p,q,\alpha }$.

#### Proof

Let *g* be elementary adapted. For $k = (k_n) \in \mathcal {K}_{\tau }$ writing $k_n = - \sum _{m=n}^\infty (k_{m+1}-k_m)$ gives

$$\begin{aligned} (I^{\tau }(k) g)_n&= - \sum _{j=0}^\infty \sum _{m=1}^\infty (k_{m+1} -k_m) \textbf{1}_{j \le n-1} \textbf{1}_{n-j \le m } \Delta _j I_g \\&= -\sum _{m=1}^\infty \sqrt{m} (k_{m+1}-k_m) \sum _{j=0}^\infty \frac{1}{\sqrt{m}} \textbf{1}_{j \le n-1} \textbf{1}_{j \ge n-m} \Delta _j I_g \\&= -\sum _{m=1}^\infty \sqrt{m\tau } (k_{m+1}-k_m) \, (J^{\tau }(m)g)_n, \end{aligned}$$

where the process $J^{\tau }(m)g :\mathbb {N}\times \Omega \rightarrow L^q(\mathcal {O})$ is given by $(J^{\tau }(m)g)_0:=0$ and for $n \ge 1$,

$$\begin{aligned} (J^{\tau }(m)g)_n :=\frac{1}{\sqrt{m\tau }} \sum _{j=0}^\infty \textbf{1}_{j \le n-1} \textbf{1}_{j \ge n-m} \Delta _j I_g = \sum _{j=0}^{n-1} k_{n-j}^{(m)} \Delta _j I_g, \end{aligned}$$

where $$k_n^{(m)} :=\frac{1}{\sqrt{m\tau }} \textbf{1}_{1\le n \le m}$$.

It follows from the above that $\mathcal {I}^{\tau }$ is contained in the closure of the absolute convex hull of $\mathcal {J}^{\tau }:=\{J^{\tau }(m):m \ge 1\}$. Therefore, by [26, Proposition 8.1.21 and Theorem 8.1.22] we see that it suffices to prove the $\mathcal {R}$-boundedness of $\mathcal {J}^{\tau }$.

Let $N \ge 1$, $a_1, \dots , a_N \in \mathbb {N}$ and $g^1, \dots , g^N \in L^p_{\mathbb {F}}(\Omega ;L^p(\mathbb {R}_+,w_{\alpha };L^q(\mathcal {O}; H)))$ be arbitrary and fixed. Note that the sequence $f^n(s) :=\Vert g^n(s)\Vert _{H}^2$ belongs to $L^{p/2}_{\mathbb {F}}(\Omega ;L^{p/2}(\mathbb {R}_+,w_{\alpha };L^{q/2}(\mathcal {O})))$.

Let $(r_n)_{n=1}^N$ be a Rademacher sequence on a probability space $(\Omega _r, {\mathscr {F}}_r, \mathbb {P}_r)$. By Proposition 2.6 applied pointwise with respect to $(\omega , m) \in \Omega _r \times \mathbb {N}$ in (i), and the Kahane–Khintchine inequalities (see [26]) in (ii) and (iii), we may estimate as follows

$$\begin{aligned}&\mathbb {E}_r \Big \Vert \sum _{n=1}^N r_n J(a_n) g^n \Big \Vert _{L^p(\Omega ; \ell ^p_{\tau ,w_{\alpha }}(L^q(\mathcal {O})))}^p \\&= \mathbb {E}_r \sum _{m \ge 1} \tau t_{m+1}^{\alpha }\,\mathbb {E}\Big \Vert \int _0^\infty \sum _{j=0}^\infty \sum _{n=1}^N \frac{r_n}{\sqrt{a_n\tau }} \textbf{1}_{ [0 \vee (m - a_n),m-1] }(j) g^n(s) \textbf{1}_{[t_j,t_{j+1})}(s) \, dW(s) \Big \Vert _{ L^q(\mathcal {O})}^p \\&\overset{(\text{ i})}{\eqsim }_{p, q} \mathbb {E}_r \sum _{m\ge 1} \tau t_{m+1}^{\alpha } \mathbb {E} \Big \Vert s \mapsto \sum _{j,n} \frac{r_n}{\sqrt{a_n \tau }} \textbf{1}_{ [0 \vee (m - a_n),m-1] }(j) g^n(s) \textbf{1}_{[t_j,t_{j+1})}(s) \Big \Vert _{L^q(\mathcal {O};L^2(\mathbb {R}_+;H))}^p \\&\overset{( \text {ii} )}{\eqsim }_{p,q} \mathbb {E} \sum _{m\ge 1} \tau t_{m+1}^{\alpha } \left( \mathbb {E}_r \Big \Vert s \mapsto \sum _{j,n} \frac{r_n}{\sqrt{a_n \tau }} \textbf{1}_{ [0 \vee (m - a_n),m-1] }(j) g^n(s) \textbf{1}_{[t_j,t_{j+1})}(s) \Big \Vert _{L^q(\mathcal {O}; L^2(\mathbb {R}_+; H))}^q \right) ^{p/q} \\&= \mathbb {E} \sum _{m\ge 1}\tau t_{m+1}^{\alpha }\, \left( \int _{\mathcal {O}} \mathbb {E}_r \Big \Vert s \mapsto \sum _{i,n} \frac{r_n}{\sqrt{a_n \tau }} \textbf{1}_{ [0 \vee (m - a_n),m-1] }(j) g^n(s) \textbf{1}_{[t_j,t_{j+1})}(s) \Big \Vert _{L^2(\mathbb {R}_+; H)}^q d\mu \right) ^{p/q} \\&\overset{(\text {iii})}{\eqsim }_q \mathbb {E} \sum _{m\ge 1}\tau t_{m+1}^{\alpha }\, \left( \int _{\mathcal {O}} \Big ( \mathbb {E}_r \Big \Vert s \mapsto \sum _{j,n} \frac{r_n}{\sqrt{a_n \tau }} \textbf{1}_{ [0 \vee (m - a_n),m-1] }(j) g^n(s) \textbf{1}_{[t_j,t_{j+1})}(s) \Big \Vert _{L^2(\mathbb {R}_+;H)}^2 \Big )^{ q/2} d\mu \right) ^{p/q} \\&= \mathbb {E} \sum _{m\ge 1}\tau t_{m+1}^{\alpha }\, \left( \int _{\mathcal {O}} \left( \sum _{j,n} \int _{t_j}^{t_{j+1}} \frac{1}{a_n \tau } \textbf{1}_{ [0 \vee (m - a_n),m-1] }(j) \Vert g^n(s)\Vert _H^2 ds \right) ^{ q/2} d\mu \right) ^{p/q} \\&\eqsim _{\alpha } \mathbb {E} \sum _{m\ge 1} \int _{t_m}^{t_{m+1} }\, \left( \int _{\mathcal {O}} \left( \sum _{n=1}^N T^*(a_n \tau ) f^n (t_m) \right) ^{ q/2} d\mu \right) ^{p/q} t^{\alpha } dt \\  &\le 2^{p/2} \mathbb {E} \sum _{m\ge 1} \int _{t_m}^{t_{m+1} }\, \left( \int _{\mathcal {O}} \left( \sum _{n=1}^N T^*((a_n+1) \tau ) f^n (t) \right) ^{ q/2} d\mu \right) ^{p/q} t^{\alpha } dt \\  &= 2^{p/2}\mathbb {E} \Big \Vert \sum _{n=1}^N T^*((a_n+1) \tau ) f^n \Big \Vert _{L^{p/2}(\mathbb {R}_+,w_{\alpha };L^{q/2}(\mathcal {O}))}^{p/2}, \end{aligned}$$

where the operator $T^*(\delta )$ on $L^{p/2}(\mathbb {R}_+,w_{\alpha };L^{q/2}(\mathcal {O}))$ is the adjoint of the one defined in Lemma 5.2 below and is given by

$$ T^*(\delta ) f (t) :=\frac{1}{\delta }\int _{(t-\delta )\vee 0}^t f(s) ds. $$

Moreover, we used the simple estimate $T^*(a_n \tau ) f (t_m)\le 2 T^*((a_n+1) \tau ) f (t)$ for all $t\in [t_m, t_{m+1})$.

Note that $w_{\alpha }\in A_{p/2}$ (see [17, Example 7.1.7]). By Lemma 5.2 with $r'=p/2$ and $s'=q/2$ we obtain

$$\begin{aligned} \Big \Vert \sum _{n=1}^N T^*((a_n+1) \tau ) f^n \Big \Vert _{L^{p/2}(\mathbb {R}_+,w_{\alpha };L^{q/2}(\mathcal {O}))}^{p/2}&\lesssim _{p,q} \Big \Vert \sum _{n=1}^N f^n \Big \Vert _{L^{p/2}(\mathbb {R}_+,w_{\alpha };L^{q/2}(\mathcal {O}))}^{p/2} \\  &\eqsim \mathbb {E}_r\Big \Vert \sum _{n=1}^N r_n g^n\Big \Vert _{L^p(\mathbb {R}_+,w_{\alpha }; L^q(\mathcal {O}; H))}^p, \end{aligned}$$

where the last step follows from reversing the computations involving the Kahane Khintchine inequalities. Combining the estimates, gives the required $\mathcal {R}$-boundedness. $\square$

In the previous proof we used a result of [46, Section 3] for the Muckenhoupt weighted setting. For details on such weights, the reader is referred to [17, Chapter 7].

#### Lemma 5.2

For $\delta >0$ let $T(\delta )$ be the operator on $L^r(\mathbb {R};L^s(\mathcal {O}))$ given by

$$\begin{aligned} T(\delta )f(t):=\frac{1}{\delta }\int _{t}^{t+\delta } f(\xi ) d\xi , \end{aligned}$$

where $r\in (1,\infty ]$ and $s\in (1, \infty )$ (where $s=\infty$ is also allowed if $r=\infty$). Let $\frac{1}{r}+\frac{1}{r'} = 1$, $\frac{1}{s}+\frac{1}{s'}=1$. Let *v* be an $A_{r'}$-weight (with $v=1$ if $r=\infty$). Then there is a constant *C* only depending on *r*, *s* and $[v]_{A_{r'}}$ such that for all $N\ge 1$, $f_1, \ldots , f_N\in L^{r'}(\mathbb {R},v;L^{s'}(\mathcal {O}))$ and $\delta _1,\ldots , \delta _N>0$,

$$\begin{aligned} \Big \Vert \sum _{n=1}^N T^*(\delta _n) f_n \Big \Vert _{L^{r'}(\mathbb {R},v;L^{s'}(\mathcal {O}))} \le C \Big \Vert \sum _{n=1}^N f_n \Big \Vert _{L^{r'}(\mathbb {R},v;L^{s'}(\mathcal {O}))}. \end{aligned}$$

To deduce the result from [46, Lemma 3.3] it suffices to note that the Hardy–Littlewood maximal operator *M* is bounded on $L^{r}(\mathbb {R},v;\ell ^{s})$ for all $v\in A_{r}$ (see [17, Theorem 7.1.9 and Corollary 7.5.7]), where $s=\infty$ is allowed if $r=\infty$. These are the weighted versions of the Fefferman-Stein maximal estimates.

A related class of operators is also $\mathcal {R}$-bounded for similar reasons.

#### Remark 5.3

For $k =(k_n)_{n\ge 1} \in \mathcal {K}_{\tau }$ and elementary adapted process $g:\mathbb {R}_+\times \Omega \rightarrow L^q(\mathcal {O};H)$ define the process $\widetilde{I}^{\tau }(k)g :\mathbb {N}\times \Omega \rightarrow L^q(\mathcal {O})$ via

$$\begin{aligned} (\widetilde{I}^{\tau }(k)g)_0 :=0 \quad \text {and } \quad (\widetilde{I}^{\tau }(k)g)_n :=\sum _{j\ge n} k_{j-n} \Delta _j I_g \quad n \ge 1 . \end{aligned}$$

Then one can show that $\widetilde{\mathcal {I}}^{\tau } = \{\widetilde{I}^{\tau }(k):k\in \mathcal {K}_{\tau }\}$ is $\mathcal {R}$-bounded by a constant only depending on *p* and *q*. Indeed, the argument can be done in a similar way as we have seen in Theorem 5.1. One difference is that one needs to reverse the roles of *T* and $T^*$.

#### Remark 5.4

The assertions of Theorem 5.1 and Remark 5.3 remain valid if $X_0$ is isomorphic to a closed subspace of $L^q(\mathcal {O})$ with $(\mathcal {O}, \Sigma ,\mu )$ a $\sigma$-finite measure space. Here one needs to replace $L^q(\mathcal {O};H)$ by $\gamma (H,X_0)$. Indeed, after applying the isomorphism, one can reduce to $L^q(\mathcal {O})$.

The technique to prove Theorem 5.1 originates from [46], but was further analyzed in [47]. Combining [46, Theorem 4.7] with the techniques in Theorem 5.1 and Remark 5.3 one can see that our results remain valid if $X_0$ is isomorphic to a Banach function space such that the 2-concavification is a UMD space. In this way, the result follows for spaces such as $L^{q}(L^{r})$, Besov spaces $B^{s}_{q,r}$, and Triebel-Lizorkin spaces $F^{s}_{q,r}$.

### Kernels for exponential schemes

In this subsection, we check that $k\in \mathcal {K}_\tau$ (as defined in Sect. 5.2) for sequences *k* which will be needed in Sect. 6. Although the proofs are completely elementary, they are quite tedious, and we give the detailed arguments for the convenience of the reader. The aim is to prove $\frac{k}{C}\in \mathcal {K}_\tau$, where $C>0$ is independent of $\tau$. In all examples, it will be obvious that $k_n\rightarrow 0$ as $n\rightarrow \infty$ and thus we only need to check that

$$\begin{aligned} \sum _{n \ge 1} \sqrt{n \tau } \, |k_{n+1}-k_n| \le C. \end{aligned}$$

This shows that $I^{\tau }(k)\in \mathcal {I}^\tau$ and $\widetilde{I}^{\tau }(k)\in \widetilde{\mathcal {I}}^\tau$, and both families are $\mathcal {R}$-bounded by Theorem 5.1 and Remark 5.3.

In the proofs below, we use the following elementary facts:

#### Lemma 5.5

Let $$\nu \in (0,\pi /2)$$. Then,

5.1 $$\begin{aligned} |1- e^{-z}|&\le |z|, \quad \text {for all }z\in \mathbb {C}\text { with }\Re (z) \ge 0, \end{aligned}$$

5.2 $$\begin{aligned} |1-e^{-z}|&\le M_{\nu }(1-e^{-|z|\cos (\arg {z})}), \quad \text {for all }z\in \Sigma _{\nu }. \end{aligned}$$

#### Proof

For real *z*, (5.1) can be proved by comparing derivatives. The complex case follows from the real case since $|1- e^{-z}| \le |z|\int _0^1 |e^{-tz}| dt = |z|\int _0^1 e^{-t \Re (z)} dt = \frac{|z|}{\Re (z)} |1- e^{-\Re (z)}|\le |z|$.

At the same time, this gives (5.2). Indeed, observe that $\Re (z) = |z| \cos (\arg (z))$ and that $\frac{|z|}{\Re (z)} = \frac{1}{\cos (\arg (z))}$. Thus, the result follows with $M_{\nu } = \frac{1}{\cos (\nu )}$. $\square$

#### Lemma 5.6

Let $\nu \in (0,\pi /2)$ and $\sigma \in (0,1/2)$. Let $k_n, \phi :(0,\infty )\times \Sigma _{\nu }\rightarrow \mathbb {C}$ be given by

$$\begin{aligned} k_n(\tau ,\lambda )&= \lambda ^{1/2} e^{-n\tau \lambda }, \ \ n\ge 1, \\ \phi (\tau , \lambda )&= \sum _{j\ge 1} \frac{e^{-j\tau \lambda } - 1}{(\tau \lambda )^{\sigma }} a_j, \end{aligned}$$

where $|a_j|\le b/j^{1+\sigma }$ for all $j\ge 1$, and $b\ge 0$ is a constant. Then there are constants $C_{\nu }$ and $C_{\nu , \sigma }$ such that for all $\tau >0$ and $\lambda \in \Sigma _{\nu }$,

$$\begin{aligned} \text {both } \ \frac{k(\tau ,\lambda )}{b C_{\nu }} \ \ \text {and} \ \ \frac{k(\tau , \lambda ) \phi (\tau , \lambda )}{b C_{\nu ,\sigma }} \ \ \text {define sequences in }\mathcal {K}_\tau . \end{aligned}$$

#### Proof

Setting $c=\cos \nu$, we see that $|e^{-n\tau \lambda }| \le e^{-c n\tau |\lambda |}$. Using this and (5.1) we find that

$$\begin{aligned} |k_{n+1}(\tau , \lambda )-k_n(\tau , \lambda )| = |\lambda |^{1/2} |e^{-n\tau \lambda }| |e^{-\tau \lambda } - 1| \le |\lambda |^{1/2} e^{-n\tau c|\lambda |} |\tau \lambda |. \end{aligned}$$

It follows that

$$\begin{aligned} \sum _{n \ge 1} \sqrt{n \tau } \, |k_{n+1}(\tau , \lambda )-k_n(\tau , \lambda )|&\le \sum _{n\ge 1 } \tau |\lambda |^{\frac{3}{2}} \sqrt{n\tau } e^{-cn\tau |\lambda |} \\  &= \sum _{n\ge 1} \int _{t_{n}}^{t_{n+1}} |\lambda |^{\frac{3}{2}} t_n^{\frac{1}{2}} e^{-ct_n |\lambda |} dt \\  &\le \int _0^\infty |\lambda |^{\frac{3}{2}} t^{\frac{1}{2}} e^{-ct |\lambda |/2} dt = c^{-3/2}\int _0^\infty s^{\frac{1}{2}} e^{-s/2} ds, \end{aligned}$$

which is a finite number only depending on $\nu$.

To prove the bound for $k(\tau ,\lambda )\phi (\tau ,\lambda )$, it remains to prove that $\phi$ is uniformly bounded. To do so, note that by (5.2)

5.3 $$\begin{aligned} \begin{aligned} |\phi (\tau , \lambda )|&\le M_{\nu } b\sum _{j\ge 1} \frac{1-e^{-j |\tau \lambda | c}}{|\tau \lambda |^{\sigma } j^{1+\sigma }} \\  &= M_{\nu } b\sum _{j\ge 1} \int _{t_j}^{t_{j+1}} \frac{1-e^{-j |\tau \lambda |c}}{|\tau \lambda |^{\sigma } j^{1+\sigma } \tau } dt \\  &\le M_{\nu } b\int _{0}^{\infty } \frac{1-e^{- t |\lambda |c }}{|\lambda |^{\sigma } (t/2)^{1+\sigma }} dt = M_{\nu }b 2^{1+\sigma } c^{\sigma } \int _{0}^{\infty } \frac{1-e^{- t}}{t^{1+\sigma }} dt, \end{aligned} \end{aligned}$$

which is a finite number only depending on $\nu$ and $\sigma$. $\square$

We will also need the following version.

#### Lemma 5.7

Let $\nu \in (0,\pi /2)$ and $\sigma \in (0,1/2)$. Let $\widetilde{k}_n:(0,\infty )\times \Sigma _{\nu }\rightarrow \mathbb {C}$ be given by

$$\begin{aligned} \widetilde{k}_n(\tau , \lambda )&= \sum _{j\ge n+1} \frac{\lambda ^{1/2}}{(\tau \lambda )^{\sigma }} e^{-(j-n)\tau \lambda } a_j, \ \ n\ge 1, \end{aligned}$$

where $(a_j)_{j\ge 1}$ is a sequence in $\mathbb {C}$ that satisfies $|a_j-a_{j+1}|\le \frac{b}{j^{2+\sigma }}$, where $b\ge 0$ is a constant. Then there is a constant $C_{\nu ,\sigma }$ such that for all $\tau >0$ and $\lambda \in \Sigma _{\nu }$,

$$\begin{aligned} \frac{\widetilde{k}(\tau , \lambda )}{b C_{\nu ,\sigma }} \ \ \text {defines a sequence in }\mathcal {K}_\tau . \end{aligned}$$

#### Proof

A rewriting gives

$$\begin{aligned}&|\widetilde{k}_{n}(\tau , \lambda )- \widetilde{k}_{n+1}(\tau , \lambda ) | \\  &\quad = \frac{|\lambda |^{1/2}}{|\tau \lambda |^{\sigma }} \Big |\sum _{j\ge n+1} e^{-(j-n)\tau \lambda } (a_{j} - a_{j+1})\Big | \\  &\quad \le \frac{|\lambda |^{1/2}}{|\tau \lambda |^{\sigma }} \sum _{j\ge n+1} e^{-(j-n)\tau |\lambda | c } \frac{b}{j^{2+\sigma }} \\  &\quad = \frac{b|\lambda |^{1/2}}{|\tau \lambda |^{\sigma }} e^{-\tau |\lambda | c } \frac{1}{(n+1)^{2+\sigma }} + b|\lambda |^{1/2-\sigma } \tau \sum _{j\ge n+2} \int _{t_{j-1}}^{t_{j}} e^{-(t_j-t_n)|\lambda | c } \frac{1}{t_j^{2+\sigma }} ds \\  &\quad \le \frac{b|\lambda |^{1/2}}{|\tau \lambda |^{\sigma }} e^{-\tau |\lambda | c } \frac{1}{(n+1)^{2+\sigma }} + b|\lambda |^{1/2-\sigma } \tau \int _{t_{n+1}}^{\infty } e^{-(s-t_n)|\lambda | c } \frac{1}{s^{2+\sigma }} ds =:A_n+B_n, \end{aligned}$$

where we took out the $j=n+1$ term because later it is helpful to start the integral at $t_{n+1}$ instead of $t_n$. Next, multiplying by $\sqrt{n\tau }$ and summing over $n\ge 1$ the $A_n$-term becomes

$$\begin{aligned} \sum _{n\ge 1} \sqrt{n\tau } A_n \le b|\tau \lambda |^{1/2-\sigma } e^{-\tau |\lambda | c } \sum _{n\ge 1} \frac{1}{(n+1)^{\frac{3}{2}+\sigma }} \le b\sup _{r>0} r^{1/2-\sigma } e^{-r c } \sum _{n\ge 1} \frac{1}{(n+1)^{\frac{3}{2}+\sigma }}, \end{aligned}$$

which is a number only depending on $\nu$ and $\sigma$. The $B_n$-term can be estimated as

$$\begin{aligned} \sum _{n\ge 1} \sqrt{n\tau } B_n&\le b|\lambda |^{1/2-\sigma } \sum _{n\ge 1} t_n^{1/2} \tau \int _{t_{n+1}}^{\infty } e^{-(s-t_n)|\lambda | c } \frac{1}{s^{2+\sigma }} ds \\  &= b|\lambda |^{1/2-\sigma } \sum _{n\ge 1} \int _{t_{n}}^{t_{n+1}} \int _{t_{n+1}}^{\infty } t_n^{1/2} e^{-(s-t_n)|\lambda | c } \frac{1}{s^{2+\sigma }} ds dt \\  &\le b|\lambda |^{1/2-\sigma } \int _{0}^{\infty } \int _{t}^{\infty } t^{1/2} e^{-(s-t)|\lambda | c } \frac{1}{s^{2+\sigma }} ds dt \\  &= b|\lambda |^{1/2-\sigma } \int _{0}^{\infty } \int _{1}^{\infty } e^{-t(u-1)|\lambda | c } \frac{1}{t^{1/2+\sigma } u^{2+\sigma }} du dt \\  &= b|\lambda |^{1/2-\sigma } \int _{0}^{\infty } \int _{0}^{\infty } e^{-t u|\lambda | c } \frac{1}{t^{1/2+\sigma } (u+1)^{2+\sigma }} dt du \\  &= b c^{\sigma -\frac{1}{2}} \int _{0}^{\infty } \frac{e^{-v}}{v^{1/2+\sigma }} dv \int _{0}^{\infty } \frac{u^{\sigma -\frac{1}{2}}}{(u+1)^{2+\sigma }} du, \end{aligned}$$

which is a constant depending only on $\nu$ and $\sigma$. $\square$

### Kernels for rational schemes

Next, we check $k\in \mathcal {K}_\tau$ for sequences associated to rational schemes. The proofs are similar to those of the exponential function, and sometimes we can rely on the latter in proving estimates.

The following assumption will be in place:

#### Assumption 5.8

Let $\theta \in (0,\pi /2]$. Let $r:\Sigma _{\theta }\rightarrow \mathbb {C}$ be a rational function such that *r* is consistent of order $\ell \ge 1$, $A(\theta )$-stable, i.e. $|r(z)|\le 1$ for $z\in \Sigma _{\theta }$, and $r(\infty ) = 0$.

Recall that this assumption is precisely what is needed for Lemma 2.2.

#### Lemma 5.9

Suppose that Assumption 5.8 holds and let $\nu \in (0,\theta )$. Let $\sigma \in (0,1/2)$. Let $k_n^r, \phi ^r:(0,\infty )\times \Sigma _{\nu }\rightarrow \mathbb {C}$ be defined by

$$\begin{aligned} k_n^r(\tau ,\lambda )&=\lambda ^{\frac{1}{2}} r(\tau \lambda )^n, \ \ n\ge 1, \\ \phi ^r(\tau , \lambda )&= \sum _{j \ge 1} \frac{r(\tau \lambda )^j-1}{(\tau \lambda )^{\sigma }} a_j, \end{aligned}$$

where $|a_j|\le b/j^{1+\sigma }$ for all $j\ge 1$, and $b\ge 0$ is a constant. Then there are constants $C_{\nu ,\theta }$ and $C_{\nu ,\theta ,\sigma }$ such that for all $\tau >0$ and $\lambda \in \Sigma _{\nu }$,

$$\begin{aligned} \text {both} \ \ \frac{k_n^r(\tau ,\lambda )}{b C_{\nu ,\theta }} \ \ \text {and} \ \ \frac{k_n^r(\tau ,\lambda ) \phi ^r(\tau , \lambda )}{b C_{\nu ,\theta ,\sigma }} \ \ \text {define sequences in }\mathcal {K}_\tau . \end{aligned}$$

#### Proof

Let $$z=\tau \lambda $$. Then

$$\begin{aligned} \sum _{n\ge 1}\sqrt{n\tau } |k_{n+1}^r(\tau , \lambda )-k_n^r(\tau , \lambda )| = |z|^{\frac{1}{2}} |r(z)-1| \sum _{n\ge 1} \sqrt{n} |r(z)|^n. \end{aligned}$$

We bound the latter uniformly in *z*. First, consider $z\in \Sigma _{\theta }$ with $|z|\ge 1$. Then by (2.11)

$$\begin{aligned} |r(z)-1| \, |z|^{\frac{1}{2}} \sum _{n\ge 1} \sqrt{n} \, |r(z)|^n \le C_1 (C_1+1) \sum _{n\ge 1} \sqrt{n} \, e^{-c_0n}, \end{aligned}$$

which is a constant only depending on $\theta$ and $\nu$.

Next, consider $z\in \Sigma _{\theta }$ with $|z|<1$. Then by (2.10) and (5.1), $|r(z)|^n \le C_1 e^{-c_0 n|z|}$, and

$$\begin{aligned} |r(z)-1|\le |r(z)-e^{-z}| + |e^{-z}-1| \le (C_1 + 1)|z|. \end{aligned}$$

Therefore,

$$\begin{aligned} |r(z)-1| \, |z|^{\frac{1}{2}} \sum _{n\ge 1} \sqrt{n} \, |r(z)|^n&\le \frac{|r(z)-1|}{|z|} \sum _{n\ge 1} \sqrt{n} |z|^{3/2} e^{-c_0n|z|}\\&\le (C_1 + 1) \sum _{n\ge 1} \sqrt{n} |z|^{3/2} e^{-c_0n|z|}, \end{aligned}$$

which can be bounded as in the proof of Lemma 5.6.

To prove the same for $k^r(\tau ,\lambda ) \phi ^r(\tau ,\lambda )$, it is enough to bound $\phi ^r$ uniformly. To do so note that

$$\begin{aligned} |\phi ^r(\tau ,\lambda )| \le b \sum _{j \ge 1} \frac{|r(z)^j-1|}{|z|^{\sigma } j^{1+\sigma }}. \end{aligned}$$

For $z\in \Sigma _{\nu }$ and $|z|\ge 1$ using that $|r(z)|\le 1$, we see that $|\phi ^r(\tau ,\lambda )| \le 2b\sum _{j \ge 1} \frac{1}{j^{1+\sigma }}$.

For $|z|<1$ note that by (2.9) and (5.2)

$$\begin{aligned} |r(z)^j-1|\le |r(z)^j-e^{-jz}|+|e^{-jz}-1|\le C_1 (j |z|^{1} e^{-c_0j|z|}+ 1- e^{-c j|z|}), \end{aligned}$$

where $$c = \cos (\nu )$$. Therefore,

$$\begin{aligned} |\phi ^r(\tau ,\lambda )| \le b C_1 \sum _{j \ge 1} \frac{|z|^{1 -\sigma } e^{-c_0j|z|}}{j^{\sigma }} + b C_1 \sum _{j \ge 1} \frac{1-e^{-c j|z|}}{|z|^{\sigma } j^{1+\sigma }}. \end{aligned}$$

The first term on the right-hand side satisfies

$$\begin{aligned} b\sum _{j \ge 1} \frac{|z|^{1 -\sigma } e^{-c_0j|z|}}{j^{\sigma }}&= b\sum _{j \ge 1} \int _{t_{j-1}}^{t_{j}} \frac{|\lambda | e^{-c_0 t_j |\lambda |}}{|\lambda |^{\sigma } t_j^{\sigma }} dt \le b\int _{0}^{\infty } \frac{|\lambda | e^{-c_0 t |\lambda |}}{|\lambda |^{\sigma } t^{\sigma }} dt\\&= b c^{\sigma -1}_0 \int _{0}^{\infty } \frac{e^{-t}}{t^{\sigma }} dt. \end{aligned}$$

The second term in the estimate for $\phi ^r$ is uniformly bounded by (5.3). $\square$

Finally, we will need the following variant of Lemma 5.7 as well.

#### Lemma 5.10

Suppose that Assumption 5.8 holds and let $\nu \in (0,\theta )$. Let $\sigma \in (0,1/2)$. Let $\widetilde{k}_n^r:(0,\infty )\times \Sigma _{\nu }\rightarrow \mathbb {C}$ be given by

$$\begin{aligned} \widetilde{k}_n^r (\tau , \lambda )&= \sum _{j\ge n+1} \frac{\lambda ^{1/2}}{(\tau \lambda )^{\sigma }} r(\tau \lambda )^{j-n} a_j, \ \ n\ge 1, \end{aligned}$$

where $(a_j)_{j\ge 1}$ in $\mathbb {C}$ satisfies $|a_j-a_{j+1}|\le \frac{b}{j^{2+\sigma }}$, where $b\ge 0$ is a constant. Then there is a constant $C_{\nu ,\theta ,\sigma }$ such that for all $\tau >0$ and $\lambda \in \Sigma _{\nu }$,

$$\begin{aligned} \frac{\widetilde{k}^r(\tau , \lambda )}{b C_{\nu ,\theta ,\sigma }} \ \ \text {defines a sequence in }\mathcal {K}_\tau . \end{aligned}$$

#### Proof

As in the proof of Lemma 5.7, using $z = \tau \lambda$ we can write

$$\begin{aligned}&|\widetilde{k}_{n}^r(\tau , \lambda ) - \widetilde{k}_{n+1}^r(\tau , \lambda ) | \\&\quad = \frac{|\lambda |^{1/2}}{|z|^{\sigma }} \Big |\sum _{j\ge n+1} r(z)^{j-n} (a_j - a_{j+1})\Big | \\  &\quad \le \frac{b|\lambda |^{1/2}}{|z|^{\sigma }} \sum _{j\ge n+1} \frac{|r(z)|^{j-n}}{{j^{2+\sigma }}} . \\  &\quad = \frac{b|\lambda |^{1/2}}{|z|^{\sigma }} |r(z)| \frac{1}{(n+1)^{2+\sigma }} + \frac{b|\lambda |^{1/2}}{|z|^{\sigma }} \sum _{j\ge n+2} \frac{|r(z)|^{j-n}}{{j^{2+\sigma }}} =:A_n + B_n. \end{aligned}$$

The $A_n$ term multiplied by $\sqrt{n\tau }$ and summed over all $n\ge 1$ gives

$$\begin{aligned} \sum _{n\ge 1} \sqrt{n\tau } A_n = b\sum _{n\ge 1} \sqrt{n \tau } \frac{|\lambda |^{1/2}}{|z|^{\sigma }} \frac{|r(z)|}{{(n+1)^{2+\sigma }}} \le b|z|^{1/2-\sigma } |r(z)| \sum _{n\ge 1} \frac{1}{{(n+1)^{\frac{3}{2}+\sigma }}}. \end{aligned}$$

For $|z|\le 1$, we have $|z|^{1/2-\sigma } |r(z)|\le 1$. For $|z|>1$, by (2.11), $|z|^{1/2-\sigma } |r(z)| \le C_1$.

It remains to bound $\sum _{n\ge 1} \sqrt{n\tau } B_n$. Note that for $|z|\ge 1$ again by (2.11)

$$\begin{aligned} \sum _{n\ge 1} \sqrt{n\tau } B_n&\le b C_1 \sum _{n\ge 1} \sqrt{n} \sum _{j\ge n+2} \frac{e^{-c_0(j-n)}}{{j^{2+\sigma }}}, \end{aligned}$$

and the latter is finite as can be seen from the proof of Lemma 5.7 by taking $\lambda = c_0/c$.

For $|z|<1$ by (2.10) $|r(z)|^{j-n} \le b C_1 e^{-c_0(j-n)|z|}$ and therefore

$$\begin{aligned} \sum _{n\ge 1} \sqrt{n\tau } B_n&\le b C_1 \sum _{n\ge 1} \sqrt{n} |z|^{1/2-\sigma } \sum _{j\ge n+2} \frac{e^{-c_0(j-n)|z|}}{{j^{2+\sigma }}}, \end{aligned}$$

which can be bounded in the same way as in Lemma 5.7. $\square$

## Discrete maximal estimates

In this section, we will consider a maximal estimate with parabolic regularization, which includes Theorem 1.4 as a special case. In this maximal estimate one needs to bound $\mathbb {E}\sup _{n\ge 1} \Vert Y_n\Vert ^p_{Z}$ (for a suitable norm $\Vert \cdot \Vert _Z$). Since *Y* does not have a martingale structure, one often cannot apply stochastic calculus techniques, and therefore it is unclear how to deal with the supremum over *n* inside an expectation. Especially, if the norm $\Vert \cdot \Vert _Z$ is chosen to express optimal parabolic regularization, this leads to serious issues. In the deterministic setting there is no expectation, so that this difficulty is less prominent. Maximal estimates in the latter case are well-known and the reader is referred to Remark 6.2 for further details.

### Maximal estimate with parabolic regularization

The following result is the main result of this section and, in particular, includes Theorem 1.4.

Recall from (3.1) that *Y* is given by the recursive formula $Y_0=0$ and

$$\begin{aligned} Y_{n+1} :=R_\tau Y_{n} + R_\tau \Delta _n I_g, \quad n\ge 0, \end{aligned}$$

where $\Delta _n I_g$ is as defined below (3.1) and $R_{\tau }$ is as in Assumption 1.1. Moreover, by (3.2),

$$ Y_n = \sum _{j=0}^{n-1} R_{\tau }^{n-j} \Delta _j I_g, \ \ n\ge 1. $$

#### Theorem 6.1

Let $q\in [2, \infty )$ and suppose that $X_0$ is isomorphic to a closed subspace of $L^q(\mathcal {O})$ with $(\mathcal {O}, \Sigma ,\mu )$ a $\sigma$-finite measure space. Suppose that Assumption 1.1 holds, that *A* has a bounded $H^\infty$-calculus on $X_0$ of angle $<\pi /2$, and that $0\in \rho (A)$. Then for any $p\in (2, \infty )$ and $\alpha \in [0,\frac{p}{2}-1)$, there is a constant *C* such that for every $g\in L^{p}_{\mathbb {F}}(\Omega ;L^p(\mathbb {R}_+,w_{\alpha };\gamma (H,X_{1/2})))$ and every stepsize $\tau >0$,

$$\begin{aligned} \mathbb {E}\sup _{n\ge 1} \Vert Y_n \Vert _{X_{1-\frac{1+\alpha }{p},p}}^p&\le C^p \mathbb {E}\Vert g\Vert ^{p}_{L^p(\mathbb {R}_+,w_{\alpha };\gamma (H,X_{1/2}))}, \\ \mathbb {E}\sup _{n\ge 1} (\tau n)^{\alpha } \Vert Y_n \Vert _{X_{1-\frac{1}{p},p}}^p&\le C^p \mathbb {E}\Vert g\Vert ^{p}_{L^p(\mathbb {R}_+,w_{\alpha };\gamma (H,X_{1/2}))}, \end{aligned}$$

where *Y* is given by (3.1).

Since $X_0$ is assumed to be isomorphic to a closed subspace of $L^q$, it follows that any of the complex interpolation spaces $X_{\beta }$ for $\beta \in [0,1]$, are isomorphic to a closed subspace of $L^q$. Indeed, since we assume that *A* has a bounded $H^\infty$-calculus it follows that $X_{\beta } = D(A^{\beta })$ isomorphically (see Lemma 2.1). Since $D(A^{\beta })$ is isomorphic to $X_0$ (use $(1+A)^{\beta }$ as an isomorphism), the result follows.

### Proof of Theorem 6.1

#### Reduction to a continuous-time setting

It is clear that by density, it suffices to consider $g\in L^p(\Omega ;L^p(\mathbb {R}_+;\gamma (H,X_{1})))$ with support on a bounded subinterval of $[0,\infty )$. Let $u \in L^p(\mathbb {R}_+\times \Omega ;X_1)$ be the linear interpolation of the discrete solution, i.e.

6.1 $$\begin{aligned} u(n\tau +s\tau )=(1-s)Y_n+sY_{n+1}, \ \ \ s \in [0,1], n \ge 0. \end{aligned}$$

From Corollary 3.9 one sees that $A Y\in L^p(\Omega ;\ell ^p_{\tau ,w_{\alpha }}(X_0))$. Moreover, since *A* is invertible we have $Y\in L^p(\Omega ;\ell ^p_{\tau ,w_{\alpha }}(X_1))$ and $u\in L^p(\Omega ;L^p(\mathbb {R}_+,w_{\alpha };X_1))$ with

6.2 $$\begin{aligned} \mathbb {E}\Vert u\Vert _{L^p(\mathbb {R}_+,w_{\alpha };X_1)}^p&\eqsim \mathbb {E}\sum _{n\ge 0} \int _{t_n}^{t_{n+1}} \Vert A u(t)\Vert ^p_{X_0} t^{\alpha } dt \\  &\nonumber = \mathbb {E}\sum _{n\ge 0} \int _{0}^{1} \Vert A u(n\tau + s\tau )\Vert ^p_{X_0} \tau (n\tau + s\tau )^{\alpha } ds \\  &\nonumber \le \mathbb {E}\sum _{n\ge 0} \int _{0}^{1} [(1-s)\Vert A Y_n\Vert ^p_{X_0}+s \Vert AY_{n+1}\Vert _{X_0}^p] \tau (n\tau + s\tau )^{\alpha } ds \\  &\nonumber \le \mathbb {E}\sum _{n\ge 0} \tau t_{n+1}^{\alpha } \Vert A Y_n \Vert ^p_{X_0} \\  &\nonumber \lesssim \mathbb {E}\Vert g\Vert _{L^p(\mathbb {R}_+,w_{\alpha };\gamma (H,X_{1/2}))}^p, \end{aligned}$$

where we also use the convexity of $|\cdot |^p$.

Since *u* is piecewise linear, one can even check that $u'$ exists in the weak sense and $u'\in L^p(\Omega ;L^p(\mathbb {R}_+;X_1))$ and also $u'\in L^p(\Omega ;L^p(\mathbb {R}_+,w_{\alpha };X_1))$ due to the extra regularity assumed on *g*. In particular, $u\in C_b(\mathbb {R}_+;X_1)$.

The proof of Theorem 6.1 would be complete if we can prove the bound

6.3 $$\begin{aligned} \mathbb {E}\sup _{t\ge 0} \Vert u(t)\Vert _{X_{1-\frac{1+\alpha }{p},p}}^p + \mathbb {E}\sup _{t\ge 0} t^{\alpha } \Vert u(t)\Vert _{X_{1-\frac{1}{p},p}}^p\le C^p \mathbb {E}\Vert g\Vert ^{p}_{L^p(\mathbb {R}_+,w_{\alpha };\gamma (H,X_{1/2}))}. \end{aligned}$$

Indeed, this is immediate from $u(t_n) = Y_n$. Now a crucial trick to avoid estimating the expectation of a supremum of a stochastic process is to use the following trace embedding of [1, Theorem 1.2] (see also [43, Theorem 1.1]): for $\sigma \in (\frac{1}{2}- \frac{1}{p}, \frac{1}{2})$

$$\begin{aligned} L^{p}(\mathbb {R}_+,w_{\alpha };X_{1}) \cap H^{\sigma ,p}(\mathbb {R}_+,w_{\alpha };X_{1-\sigma })&\hookrightarrow C_b([0,\infty );X_{1-\frac{1+\alpha }{p},p}), \\ L^{p}(\mathbb {R}_+,w_{\alpha };X_{1}) \cap H^{\sigma ,p}(\mathbb {R}_+,w_{\alpha };X_{1-\sigma })&\hookrightarrow C_b((0,\infty ),w_{\alpha };X_{1-\frac{1}{p},p}), \end{aligned}$$

where $C_b((0,\infty ),w_{\alpha };E)$ are the continuous functions $f:(0,\infty )\rightarrow E$ for which $\Vert f\Vert _{C_b((0,\infty ),w_{\alpha };E)} :=\sup _{t>0} w_{\alpha }(t) \Vert f(t)\Vert _E<\infty$.

Indeed, (6.3) follows as soon as we have proved the estimate

6.4 $$\begin{aligned} \mathbb {E}\Vert u\Vert _{H^{\sigma ,p}(\mathbb {R}_+;X_{1-\sigma })}^p \le C^p_{\sigma } \mathbb {E}\Vert g\Vert ^{p}_{L^p(\mathbb {R}_+;\gamma (H,X_{1/2}))}, \ \ \sigma \in [0,1/2). \end{aligned}$$

The case $\sigma = 0$ follows from (6.2). From now on let $\sigma \in (0,1/2)$ be arbitrary. To prove (6.4), due to the fact that $0\in \rho (A)$ it is enough to show that

$$\begin{aligned} \mathbb {E}\Vert A^{1-\sigma } u\Vert _{H^{\sigma ,p}(\mathbb {R}_+,w_{\alpha };X_{0})}^p\le C^p_{\sigma } \mathbb {E}\Vert g\Vert ^{p}_{L^p(\mathbb {R}_+,w_{\alpha };\gamma (H,X_{1/2}))}, \ \ \ \sigma \in [0,1/2). \end{aligned}$$

It is well-known that $-\partial _t$ on $L^p(\mathbb {R}_+,w_{\alpha };X_0)$ with domain $W^{1,p}(\mathbb {R}_+,w_{\alpha };X_0)$ has a bounded $H^\infty$-calculus of angle $\pi /2$ and $D(\partial _t^{\sigma }) = H^{\sigma ,p}(\mathbb {R}_+,w_{\alpha },X_0)$ (see [39, Theorem 6.8]). In particular, it follows that

$$\begin{aligned} \Vert A^{1-\sigma } u\Vert _{H^{\sigma ,p}(\mathbb {R}_+,w_{\alpha };X_{0})}\eqsim \Vert A^{1-\sigma } u\Vert _{L^{p}(\mathbb {R}_+,w_{\alpha };X_{0})} + \Vert (-\partial _t)^{\sigma } A^{1-\sigma } u\Vert _{L^{p}(\mathbb {R}_+,w_{\alpha };X_{0})}. \end{aligned}$$

Since we have already estimated the $L^p$-norm of $\Vert A^{1-\sigma } u\Vert _{X_0}\lesssim \Vert A u\Vert _{X_0}$, it remains to prove

6.5 $$\begin{aligned} \mathbb {E}\Vert (-\partial _t)^{\sigma } A^{1-\sigma } u\Vert _{L^p(\mathbb {R}_+,w_{\alpha };X_0)}^p\lesssim \mathbb {E}\Vert g\Vert ^{p}_{L^p(\mathbb {R}_+,w_{\alpha };\gamma (H,X_{1/2}))}. \end{aligned}$$

Note that $\partial _t$ generates the left translation semigroup on $L^p(\mathbb {R}_+;X_0)$. Since $A^{1-\sigma } u$ is in $W^{1,p}(\mathbb {R}_+,w_{\alpha };X_0)$, it follows from the Balakrishnan formula for the fractional power (see [42, Theorem 3.2.2]) that

6.6 $$\begin{aligned} (-\partial _t)^\sigma A^{1-\sigma } u(t) = C_{\sigma } \int _0^\infty A^{1 -\sigma } \frac{u(t)-u(t+h)}{h^{1+\sigma }} \, dh, \end{aligned}$$

with $$C_{\sigma } \ne 0$$.

#### Estimating the fractional derivative

By (6.6) we can write

$$\begin{aligned} \mathbb {E}&\Vert A^{1-\sigma } (-\partial )^\sigma u\Vert ^p_{L^p(\mathbb {R}_+,w_{\alpha };X_0)} \eqsim \mathbb {E}\int _0^\infty \Big \Vert \int _0^\infty A^{1 -\sigma } \frac{u(t)-u(t+h)}{h^{1+\sigma }} \, dh \Big \Vert ^p_{X_0} w_{\alpha }(t) \, dt \\&= \mathbb {E}\sum _{n\ge 0} \int _{t_n}^{t_{n+1}} \Big \Vert \sum _{j\ge 0} \int _{t_j}^{t_{j+1}} A^{1 -\sigma } \frac{u(t)-u(t+h)}{h^{1+\sigma }} \, dh \Big \Vert ^p_{X_0} t^{\alpha } \, dt \\&\le \mathbb {E}\sum _{n\ge 0} \tau \int _{0}^{1} \Big \Vert \sum _{j\ge 0} \tau \int _{0}^{1} A^{1 -\sigma } \frac{u(n\tau +s\tau )-u((n+j)\tau +(s+r)\tau )}{(j\tau +r\tau )^{1+\sigma }} \, dr \Big \Vert ^p_{X_0} t_{n+1}^{\alpha }\, ds. \end{aligned}$$

For $s+r\in [0,1)$ we can write

$$\begin{aligned} u(n\tau +&s\tau )-u((n+j)\tau +(s+r)\tau ) \\  &= (1-s)Y_n+sY_{n+1}-[(1-(s+r))Y_{n+j}+(s+r)Y_{n+j+1}] \\  &=(1-s-r) (Y_n-Y_{n+j}) + (s+r)(Y_{n+1}-Y_{n+j+1}) + r(Y_{n}-Y_{n+1}), \end{aligned}$$

For $s+r\in [1, 2)$ we can write

$$\begin{aligned} u(&n\tau +s\tau )-u((n+j)\tau +(s+r)\tau ) \\  &= (1-s)Y_n+sY_{n+1}-[(1-(s+r-1))Y_{n+j+1}+(s+r-1)Y_{n+j+2}] \\  &= (2-s-r)(Y_n - Y_{n+j+1}) + (s+r-1)(Y_{n+1}-Y_{n+j+2}) +(1-r)(Y_{n+1}-Y_{n}). \end{aligned}$$

Therefore, from the triangle inequality, it follows that it suffices to estimate each of the following:

$$\begin{aligned} T_{1}&:=\sum _{n\ge 0} \tau \int _0^1 \mathbb {E}\Big \Vert \sum _{j \ge 1} \int _0^{1-s} \tau A^{1 - \sigma } (1-s-r) \frac{Y_n-Y_{n+j}}{(j\tau +r\tau )^{1+\sigma }} \, dr \Big \Vert ^p_{X_0} t_{n+1}^{\alpha } \, ds \\ T_{2}&:=\sum _{n\ge 0} \tau \int _0^1 \mathbb {E}\Big \Vert \sum _{j \ge 1} \int _0^{1-s} \tau A^{1 - \sigma } (s+r)\frac{Y_{n+1}-Y_{n+j+1}}{(j\tau +r\tau )^{1+\sigma }} \, dr \Big \Vert ^p_{X_0} t_{n+1}^{\alpha }\, ds \\ T_{3}&:=\sum _{n\ge 0} \tau \int _0^1 \mathbb {E}\Big \Vert \sum _{j \ge 0} \int _0^{1-s} \tau A^{1 - \sigma } r\frac{Y_n-Y_{n+1}}{(j\tau +r\tau )^{1+\sigma }} \, dr \Big \Vert ^p_{X_0} t_{n+1}^{\alpha }ds \\ T_{4}&:=\sum _{n\ge 0} \tau \int _0^1 \mathbb {E}\Big \Vert \sum _{j \ge 1} \int _{1-s}^1 \tau A^{1 - \sigma } (2-s-r)\frac{Y_n-Y_{n+j+1}}{(j\tau +r\tau )^{1+\sigma }} \, dr \Big \Vert ^p_{X_0} t_{n+1}^{\alpha }\, ds \\ T_{5}&:=\sum _{n\ge 0} \tau \int _0^1 \mathbb {E}\Big \Vert \sum _{j \ge 0} \int _{1-s}^1 \tau A^{1 - \sigma } (s+r-1)\frac{Y_{n+1}-Y_{n+j+2}}{(j\tau +r\tau )^{1+\sigma }} \, dr \Big \Vert ^p_{X_0} t_{n+1}^{\alpha }\, ds \\ T_{6}&:=\sum _{n\ge 0} \tau \int _0^1 \mathbb {E}\Big \Vert \sum _{j \ge 1} \int _{1-s}^{1} \tau A^{1 - \sigma } (1-r)\frac{Y_{n+1}-Y_{n}}{(j\tau +r\tau )^{1+\sigma }} \, dr \Big \Vert ^p_{X_0} t_{n+1}^{\alpha }\, ds \\ T_7&:=\sum _{n\ge 0} \tau \int _0^1 \mathbb {E}\Big \Vert \int _{1-s}^{1} \tau A^{1 - \sigma } (1-s)\frac{Y_{n+1}-Y_{n}}{(r\tau )^{1+\sigma }} \, dr\Big \Vert ^p_{X_0} t_{n+1}^{\alpha } \, ds. \end{aligned}$$

The terms $T_1, T_2, T_3$ come from the case $s+r\in [0,1)$, and the other terms from $s+r\in [1,2)$. Note that the term $T_7$ is just the $j=0$ term of $T_4$ and $T_6$ combined to avoid the creation of a singularity. The proofs of the estimates for $T_1, T_2, T_4, T_5$ are very similar. To give the bounds, we only present the details for $T_1$. It is enough to prove that for all $s\in (0,1)$:

6.7 $$\begin{aligned} T_{1,s}&:=\sum _{n\ge 0} \tau \mathbb {E}\Big \Vert \sum _{j \ge 1} \tau ^{-\sigma } \psi (j,s) A^{1 - \sigma } (Y_{n+j}-Y_n) \Big \Vert ^p_{X_0}\le C\Vert g\Vert _{L^p(\mathbb {R}_+;\gamma (H,X_{1/2}))}, \end{aligned}$$

where *C* is independent of *s* and

$$\begin{aligned} \psi (j,s)&= \int _0^{1-s} \frac{(1-s-r)}{(j+r)^{1+\sigma }} \,dr. \end{aligned}$$

We need two properties of $\psi$, which are both straightforward to check:

6.8 $$\begin{aligned} \sup _{j\ge 1, s\in (0,1)} j^{1+\sigma } |\psi (j,s)|<\infty \ \ \text {and} \ \ \sup _{j\ge 1, s\in (0,1)} j^{2+\sigma } \Big |\psi (j+1,s) - \psi (j,s)\Big |<\infty . \end{aligned}$$

The details of (6.7) will be given in Sect. 6.2.4. The proofs are based on the $H^\infty$-calculus of *A* and the $\mathcal {R}$-boundedness results provided in Sect. 5.

#### Estimating terms $T_3, T_6$ and $T_7$

To estimate $T_7$ note that

$$\begin{aligned}&\sum _{n\ge 0} \tau \int _0^1 \mathbb {E}\Big \Vert \int _{1-s}^{1} \tau A^{1 - \sigma } (1-s)\frac{Y_{n+1}-Y_{n}}{(r\tau )^{1+\sigma }} \, dr\Big \Vert ^p_{X_0} t_{n+1}^{\alpha } \, ds \\&\quad \lesssim \sum _{n\ge 0} \tau \mathbb {E}\Big \Vert \tau ^{-\sigma } A^{1 - \sigma } (Y_{n+1}-Y_{n}) \, \Big \Vert ^p_{X_0} t_{n+1}^{\alpha }. \end{aligned}$$

Before we continue, we first note that for $T_3$ and $T_6$ one has

$$\begin{aligned} C_3&:=\sum _{j\ge 0} \int _0^{1-s} \frac{r}{(j+r)^{1+\sigma }} \,dr\le \int _0^{1} \frac{1}{r^{\sigma }} \,dr+\sum _{j\ge 1} \frac{1}{j^{1+\sigma }} \,dr<\infty , \\ C_6&:=\sum _{j\ge 1} \int _{1-s}^1 \frac{1-r}{(j+r)^{1+\sigma }} \,dr\le \sum _{j\ge 1} \frac{1}{j^{1+\sigma }} \,dr<\infty \end{aligned}$$

and therefore,

6.9 $$\begin{aligned} T_{3} \le C_3^p \sum _{n\ge 0} \tau \mathbb {E}\Big \Vert \tau ^{-\sigma } A^{1 - \sigma } (Y_{n+1}-Y_{n})\Big \Vert ^p_{X_0} t_{n+1}^{\alpha } . \end{aligned}$$

The same estimate holds for $T_6$. Therefore, in order to bound $T_3, T_6, T_7$, it suffices to bound the right-hand side of (6.9). From the formula for *Y* given in (3.1), we see that

$$\begin{aligned} Y_{n+1}-Y_n= R_\tau \Delta _n I_g+\sum _{m=0}^{n-1} (R_\tau -I)R_\tau ^{n-m}\Delta _m I_g. \end{aligned}$$

Thus it suffices to bound each of the terms

$$\begin{aligned} T_{8}&:=\sum _{n\ge 0} \tau \mathbb {E}\Big \Vert \tau ^{-\sigma } A^{1-\sigma } R_{\tau } \Delta _n I_g \Big \Vert ^p_{X_0} t_{n+1}^{\alpha }, \\ T_{9}&:=\sum _{n\ge 1} \tau \mathbb {E}\Big \Vert \tau ^{-\sigma } \sum _{m=0}^{n-1} A^{1-\sigma } (R_{\tau }-I)(R_{\tau }^{n-m}) \Delta _m I_g \Big \Vert ^p_{X_0} t_{n+1}^{\alpha }. \end{aligned}$$

By Proposition 2.5 and (2.13) we can estimate

$$\begin{aligned} T_{8}&\lesssim _{p,X_0} \sum _{n\ge 0} \tau \mathbb {E}\Big \Vert \tau ^{-\sigma } A^{1-\sigma } R_{\tau } g \Big \Vert ^p_{L^2(t_n, t_{n+1};\gamma (H,X_0))} t_{n+1}^{\alpha } \\&\lesssim \sum _{n\ge 0} \tau \mathbb {E}\tau ^{-p/2} \Vert A^{1/2}g \Vert ^p_{L^2((t_n, t_{n+1});\gamma (H,X_{0}))} t_{n+1}^{\alpha } \\  &\lesssim \sum _{n\ge 0} \mathbb {E}\Vert A^{1/2}g \Vert ^p_{L^p((t_n, t_{n+1}), w_{\alpha };\gamma (H,X_{0}))} \lesssim \mathbb {E}\Vert g\Vert ^p_{L^p(\mathbb {R}_+,w_{\alpha };\gamma (H,X_{1/2}))}, \end{aligned}$$

where in the penultimate step we used Hölder’s inequality and in the last step we used Lemma 2.1. By Proposition 2.5 and Lemma 2.3 (writing $R_{\tau }-I = (R_{\tau }-e^{-\tau A}) + (e^{-\tau A}-I)$ and using (2.5)) we get that

$$\begin{aligned} T_{9}&= \sum _{n\ge 1} \tau \mathbb {E}\Big \Vert \tau ^{-\sigma } \sum _{m=0}^{n-1} A^{-(\sigma +\frac{1}{2})} (R_{\tau }-I) A^{1+\frac{1}{2}}R_{\tau }^{n-m} \Delta _m I_g \Big \Vert ^p_{X_0} t_{n+1}^{\alpha } \\&\lesssim _{p,X} \sum _{n\ge 1} \tau \mathbb {E}\Big ( \sum _{m=0}^{n-1} \Big \Vert \tau ^{-\sigma } A^{-(\sigma +\frac{1}{2})} (R_{\tau }-I) AR_{\tau }^{n-m} A^{\frac{1}{2}} g\Big \Vert ^2_{L^2(t_m, t_{m+1};\gamma (H,X_{0}))} \Big )^{p/2} t_{n+1}^{\alpha } \\&\le \sum _{n\ge 1} \tau \mathbb {E}\Big ( \sum _{m=0}^{n-1} \Vert \tau ^{-\sigma } A^{-(\sigma +\frac{1}{2})}(R_{\tau }-I) \Vert ^2_{\mathcal {L}(X_0)} \Vert AR_{\tau }^{n-m}\Vert ^2_{\mathcal {L}(X_0)} \Vert A^{\frac{1}{2}} g \Vert ^2_{L^2(t_m, t_{m+1};\gamma (H,X_{0}))} \Big )^{p/2} t_{n+1}^{\alpha } \\&\lesssim \sum _{n\ge 0} \tau \mathbb {E}\Big ( \sum _{m=0}^{n-1} \tau \frac{1}{(\tau (n-m))^{2}} \Vert g\Vert ^2_{L^2(t_m, t_{m+1};\gamma (H,X_{1/2})} \Big )^{p/2} t_{n+1}^{\alpha } \\&{\mathop {\lesssim }\limits ^{\text {(i)}}}\sum _{n\ge 0} \mathbb {E}\Big ( \sum _{m=0}^{n-1} \frac{1}{(n-m)^2} \Vert g\Vert ^2_{L^p(t_m, t_{m+1}, w_{\alpha };\gamma (H,X_{1/2})} t_{m+1}^{-2\alpha /p}\Big )^{p/2} t_{n+1}^{\alpha } \\  &= \mathbb {E}\sum _{n\ge 0} \Big ( \sum _{m=0}^{n-1} G_m^2 K(n,m) \Big )^{p/2} {\mathop {\lesssim }\limits ^{\text {(ii)}}}\mathbb {E}\sum _{m\ge 0} G_m^p = \mathbb {E}\Vert g\Vert ^p_{L^p(\mathbb {R}_+,w_{\alpha };\gamma (H,X_{1/2}))}, \end{aligned}$$

where in (i) we used Hölder’s inequality and in (ii) we used Schur’s lemma (see [18, Appendix A.2]) in $\ell ^{p/2}$ for the non-negative kernel $K(n,m) :=\frac{1}{(n-m)^2} \big (\frac{n+1}{m+1}\big )^{2\alpha /p} \textbf{1}_{0\le m <n}$ with sequences $u_n=v_n=(n+1)^{-\frac{1}{(\frac{p}{2})(\frac{p}{2})'}}$, noting that $0\le \alpha <\frac{p}{2}-1$, and where we set $G_m :=\Vert g\Vert _{L^p(t_m, t_{m+1}, w_{\alpha };\gamma (H,X_{1/2}))}$.

#### Estimating the main term $T_{1,s}$

In this section, we prove the estimate (6.7). For this, we use the operator-valued $H^\infty$-calculus. This method was first used to establish stochastic maximal $L^p$-regularity in continuous-time in [48].

We write $R_\tau = r(\tau A)$ where *r*(*z*) is either the exponential function or a rational function as in Assumption 1.1. Fix $\nu \in (\omega (A),\theta )$. In order to rewrite $T_{1,s}$, defined in (6.7), note that

$$\begin{aligned}&Y_{n+j}-Y_n = \sum _{m=n}^{n+j-1} r(\tau A)^{n+j-m}\Delta _m I_g +\sum _{m=0}^{n-1} (R_{\tau }^j-I)r(\tau A)^{n-m}\Delta _m I_g \end{aligned}$$

and thus moving part of the power of *A* to the process *g*, and using a functional calculus representation (see [26, 27]), we can write

$$\begin{aligned}&A^{1-\sigma }(Y_{n+j}-Y_n) \\&\quad = \sum _{m=n}^{n+j-1} A^{1/2-\sigma } R_{\tau }^{n+j-m} \Delta _m I_{A^{1/2}}g +\sum _{m=0}^{n-1} A^{1/2-\sigma }(R_{\tau }^j-I)R_{\tau }^{n-m}\Delta _m I_{A^{1/2}g} \\  &\quad = \frac{1}{2\pi i} \int _{\partial \Sigma _{\nu }} \left[ \sum _{m=n}^{n+j-1}f_{j,m,n}^{(1)} (\lambda ) R(\lambda ,A) \Delta _m I_{A^{1/2}g} + \sum _{m=0}^{n-1} f_{j,m,n}^{(2)}(\lambda )R(\lambda ,A) \Delta _m I_{A^{1/2}g}\right] d\lambda , \end{aligned}$$

where $f_{j,m,n}^{(1)}, f_{j,m,n}^{(2)}:\Sigma _{\nu }\rightarrow \mathbb {C}$ are given by

$$\begin{aligned} f_{j,m,n}^{(1)}(\lambda )&:=\lambda ^{1/2-\sigma } r(\tau \lambda )^{n+j-m}, \\ f_{j,m,n}^{(2)}(\lambda )&:=\lambda ^{1/2-\sigma }(r(\tau \lambda )^j-I)r(\tau \lambda )^{n-m}. \end{aligned}$$

Therefore, to estimate $T_{1,s}$ it suffices to bound $T_{1,s}^{(1)}$ and $T_{1,s}^{(2)}$, which are given by

$$\begin{aligned} T_{1,s}^{(1)}&= \mathbb {E}\sum _{n\ge 0} \tau \Big \Vert \int _{\partial \Sigma _\nu } \left[ \sum _{j \ge 1} \tau ^{-\sigma } \psi (j,s) \sum _{m=n}^{n+j-1} f_{j,m,n}^{(1)}(\lambda ) R(\lambda ,A) \Delta _m I_{A^{1/2}g}\right] d\lambda \Big \Vert _{X_0}^p, \\ T_{1,s}^{(2)}&= \mathbb {E}\sum _{n\ge 0} \tau \Big \Vert \int _{\partial \Sigma _\nu } \left[ \sum _{j \ge 1} \tau ^{-\sigma } \psi (j,s) \sum _{m=0}^{n-1} f_{j,m,n}^{(2)}(\lambda ) R(\lambda ,A) \Delta _m I_{A^{1/2}g}\right] d\lambda \Big \Vert _{X_0}^p. \end{aligned}$$

To rewrite $T_{1,s}^{(1)}$ in the form of a discrete convolution operator, note that for $h\in L^{p}_{\mathbb {F}}(\Omega ;L^p(\mathbb {R}_+;\gamma (H,X_{0})))$,

$$\begin{aligned}&\sum _{j \ge 1} \tau ^{-\sigma } \psi (j,s) \sum _{m=n}^{n+j-1} f_{j,m,n}^{(1)}(\lambda ) \Delta _m I_{h} \\&\quad = \sum _{m\ge n} \sum _{j\ge m-n+1} \psi (j,s) \tau ^{-\sigma } \lambda ^{1/2-\sigma } r(\tau \lambda )^{n+j-m} \Delta _m I_{h} \\  &\quad = \sum _{m\ge n} k_{m-n}^{(1)}(\lambda ) \Delta _m I_{h} = \widetilde{I}^{\tau }(k^{(1)}(\lambda )) h, \end{aligned}$$

where

$$\begin{aligned} k_m^{(1)}(\lambda ) = \sum _{j\ge m+1} \psi (j,s) \tau ^{-\sigma } \lambda ^{1/2-\sigma } r(\tau \lambda )^{j-m}, \end{aligned}$$

and $\widetilde{I}^{\tau }(k(\lambda ))$ is as in Remark 5.3. We did not make the $\tau$-dependence explicit in *k*, but of course it also depends on $\tau$. It follows that $T_{1,s}^{(1)}$ can be written as

$$\begin{aligned} T_{1,s}^{(1)}&= \Big \Vert \int _{\partial \Sigma _\nu } \widetilde{I}^{\tau }(k^{(1)}(\lambda )) R(\lambda ,A) A^{1/2}g d\lambda \Big \Vert ^p_{L^p(\Omega ;\ell ^p_{\tau ,w_{\alpha }}(X_0))}. \end{aligned}$$

Let us introduce the short-hand notation

$$\begin{aligned} \mathcal {L}^\tau :=\mathcal {L}(L^{p}_{\mathbb {F}}(\Omega ;L^p(\mathbb {R}_+,w_{\alpha };\gamma (H,X_{0}))), L^p(\Omega ;\ell ^p_{\tau ,w_{\alpha }}(X_0))). \end{aligned}$$

One can check that the operator-valued function $\lambda \mapsto \widetilde{I}^{\tau }(k^{(1)}(\lambda ))$ is in $H^1(\Sigma _\theta ; \mathcal {L}^\tau )$, and commutes with the resolvent of *A* (seen as an operator on $L^p(\Omega ;\ell ^p_{\tau ,w_{\alpha }}(X_0))$). We claim that the above operator-valued function as a family in $\mathcal {L}^\tau$ has $\mathcal {R}$-bounded range (with uniform estimates in *s* and $\tau$). As soon as we have checked this, it follows from the boundedness of the $H^\infty$-calculus of *A* and [27, Theorem 16.3.4] that

$$\begin{aligned} T_{1,s}^{(1)}\le C \Vert A^{1/2} g\Vert _{L^{p}(\Omega ;L^p(\mathbb {R}_+;\gamma (H,X_{0})))} \lesssim \Vert g\Vert _{L^{p}(\Omega ;L^p(\mathbb {R}_+,w_{\alpha };\gamma (H,X_{1/2})))}, \end{aligned}$$

where *C* does not depend on *s* and $\tau$, and where in the last step we used Lemma 2.1.

To prove the claim, note that by Remark 5.3 it suffices to show that there is a constant *C* independent of $\lambda$ and *s* such that $k^{(1)}(\lambda )/C$ is in $\mathcal {K}_{\tau }$. This follows from Lemmas 5.7, 5.10 and (6.8).

A similar argument can be used to estimate $T_{1,s}^{(2)}$. Indeed,

$$\begin{aligned} \sum _{j \ge 1} \tau ^{-\sigma } \psi (j,s) \sum _{m=0}^{n-1} f_{j,m,n}^{(2)}(\lambda ) \Delta _m I_{h}&= \sum _{m=0}^{n-1} \sum _{j \ge 1} \tau ^{-\sigma } \psi (j,s) f_{j,m,n}^{(2)}(\lambda ) \Delta _m I_{h} \\  &= \sum _{m=0}^{n-1} k_{n-m}^{(2)}(\lambda ) \Delta _m I_{h} = I^{\tau }(k^{(2)}(\lambda )) h, \end{aligned}$$

where

$$k_m^{(2)}(\lambda ) = \sum _{j \ge 1} \tau ^{-\sigma } \psi (j,s) \lambda ^{1/2-\sigma }(r(\tau \lambda )^j-I)r(\tau \lambda )^{m},$$

and $I_\tau (k(\lambda ))$ is as in Sect. 5.2. It follows that $T_{1,s}^{(2)}$ can be written as

$$\begin{aligned} T_{1,s}^{(2)}&= \Big \Vert \int _{\partial \Sigma _\nu } I^{\tau }(k^{(2)}(\lambda )) R(\lambda ,A) A^{1/2}g d\lambda \Big \Vert ^p_{L^p(\Omega ;\ell ^p_{\tau ,w_{\alpha }}(X_0))}. \end{aligned}$$

Now the proof can be completed as we did for $T_{1,s}^{(1)}$, where this time the $\mathcal {R}$-boundedness of the range of $\lambda \mapsto I^{\tau }(k^{(2)}(\lambda ))$ follows from Theorem 5.1, Lemmas 5.6, 5.9 and (6.8).

##### Remark 6.2

In the deterministic setting, maximal estimates in trace spaces are well-known, and can be found in [9, Theorem 2.3.2] and [32, Lemma 14]. The deterministic setting is much simpler because one can formulate an equivalent description of the trace norm in a discrete setting. Moreover, expectations do not play any role here.

Our argument for proving maximal estimates can also be used to give an alternative proof for the deterministic analogue. Indeed, typically, discrete maximal regularity in the deterministic setting involves the quantity

$$\begin{aligned} \Vert D_{\tau }Y\Vert _{\ell ^p_{\tau }(X_0)} + \Vert Y\Vert _{\ell ^p_{\tau }(X_1)}, \end{aligned}$$

where $(D_{\tau }Y)_n = \frac{Y_{n+1}-Y_n}{\tau }$. Extending *Y* to a continuous-time function *u* as in (6.1) immediately gives $u\in W^{1,p}(\mathbb {R}_+;X_0)\cap L^p(\mathbb {R}_+;X_1)$, and thus the trace regularity $u\in C_b([0,\infty );X_{1-\frac{1}{p},p})$ follows from the classical Lions–Peetre trace method for real interpolation (see [27, Appendix L]).

### Proof of the maximal estimate of Proposition 1.5

We will actually prove the following more general result, which reduces to Proposition 1.5 if $s=2$.

#### Proposition 6.3

Let $X_0$ be a Hilbert space. Suppose that Assumption 1.1 holds with $\theta =\pi /2$. Suppose that $0\in \rho (A)$ or that *A* has a bounded $H^\infty$-calculus. Then for every $s\in (0,\infty )$ there is a constant *C* such that for every $g\in L^{s}_{\mathbb {F}}(\Omega ;L^2(\mathbb {R}_+;\gamma (H,X_{1/2})))$ and every stepsize $\tau >0$,

$$\begin{aligned} \mathbb {E}\sup _{n\ge 1} \Vert Y_n \Vert _{X_{1/2}}^s \le C^s \mathbb {E}\Vert g\Vert ^{s}_{L^2(\mathbb {R}_+;\gamma (H,X_{1/2}))}, \end{aligned}$$

where $Y=(Y_n)_{n\ge 0}$ is given by (1.1).

#### Proof

Note that $X_{1/2} = (X_0, X_1)_{1/2,2}$ and $\gamma (H,X_{1/2}) = \mathcal {L}_2(H,X_{1/2})$ with equivalent norms (see [25, Corollary C.4.2] and [26, Proposition 9.1.9]). If $0\in \rho (A)$, then *A* has a bounded $H^\infty$-calculus on $(X_0, X_1)_{1/2,2}$ by Dore’s theorem (see [27, Corollary 16.3.23]). If *A* has a bounded $H^\infty$-calculus on $X_0$, then this holds even without the condition $0\in \rho (A)$ by interpolation. From [35, Theorem 11.13] it follows that there is a Hilbert space norm $\Vert \cdot \Vert _Z$ which is equivalent to $X_{1/2}$ under which $(e^{-tA})_{t\ge 0}$ is a contraction semigroup. Let $K_1,K_2>0$ be such that $K_1\Vert x\Vert _{Z} \le \Vert x\Vert _{X_{1/2}} \le K_2\Vert x\Vert _{Z}$. Note that since $|r(z)|\le 1$ on $\Sigma _{\pi /2}$, there exists $\sigma >\pi /2$ small enough such that $r \in H^\infty (\Sigma _\sigma )$. Hence, from [26, Theorem 10.2.24] it follows that $R_{\tau }$ is contractive on *Z*.

Next, we extend the discrete dilation argument in [33, Proposition 5.1] to our setting. By the Sz.-Nagy dilation theorem [52, Theorem I.4.2] we can find a Hilbert space $\widetilde{Z}$, a contractive injection $Q:Z\rightarrow \widetilde{Z}$, a contractive projection $P:\widetilde{Z}\rightarrow Z$ , and a unitary operator $\widetilde{R}_\tau$ on $\widetilde{Z}$ such that

$$\begin{aligned} R_\tau ^j =P \widetilde{R}_\tau ^j Q, \ \ \ j\ge 0. \end{aligned}$$

It follows that

$$\begin{aligned} \Vert Y_n\Vert _{X_{1/2}}&\le K_2 \Vert Y_n\Vert _{Z} = K_2\Big \Vert \sum _{j=0}^{n-1} R_\tau ^{n-j} \, \Delta _j I_g \Big \Vert _{Z} \le K_2\Big \Vert \sum _{j=0}^{n-1} \widetilde{R}_\tau ^{-j} Q \Delta _j I_g \Big \Vert _{\widetilde{Z}}, \end{aligned}$$

where we used that $\widetilde{R}_\tau$ is unitary. Therefore, by Proposition 2.5 we obtain

$$\begin{aligned} \mathbb {E}\sup _{n\ge 1}\Vert Y_n\Vert _{X_{1/2}}^s&\le K_2^s \mathbb {E}\sup _{n\ge 1} \Big \Vert \sum _{j=0}^{n-1} \widetilde{R}_\tau ^{-j} Q \Delta _j I_g \Big \Vert _{\widetilde{Z}}^s \\  &\le K_2^s C_{s,X_0}^s \mathbb {E}\Big (\sum _{j\ge 0} \int _{t_j}^{t_{j+1}} \Vert \widetilde{R}_\tau ^{-j} Q g(t)\Vert _{\mathcal {L}_2(H,\widetilde{Z})}^2 dt\Big )^{s/2} \\  &\le K_2^s C_{s,X_0}^s \mathbb {E}\Big (\sum _{j\ge 0} \int _{t_j}^{t_{j+1}} \Vert g(t)\Vert _{\mathcal {L}_2(H,Z)}^2 dt\Big )^{s/2} \\  &\le K_1^{-s} K_2^s C_{s,X_0}^s \mathbb {E}\Vert g\Vert _{L^2(\mathbb {R}_+;\mathcal {L}_2(H,X_{1/2}))}^s. \end{aligned}$$

$$\square $$

#### Remark 6.4

In the above, it is enough to find an equivalent norm in which $R_{\tau }$ is a contraction.

1. In case $s=2$ and $R_{\tau }$ is a contraction on a Hilbert space, then using Doob’s maximal inequality for second moments and the above method, one can check that
  $$\begin{aligned} \mathbb {E}\sup _{n\ge 1} \Vert Y_n \Vert _{X_{1/2}}^2 \le 4 \mathbb {E}\Vert g\Vert ^{2}_{L^2(\mathbb {R}_+;\gamma (H,X_{1/2}))}. \end{aligned}$$
2. In case $R_{\tau }$ is a contraction on a 2-smooth Banach space $X_{1/2}$, a similar result as in Proposition 6.3 was proved in [44, Proposition 5.4].

## Data Availability

Data sharing is not applicable to this article as no new data were created or analyzed in this study.
